# Recapitulating kidney development in vitro by priming and differentiating mouse embryonic stem cells in monolayers

**DOI:** 10.1038/s41536-020-0092-5

**Published:** 2020-04-20

**Authors:** Theresa Chow, Frances T. M. Wong, Claudio Monetti, Andras Nagy, Brian Cox, Ian M. Rogers

**Affiliations:** 1 Lunenfeld Tanenbaum Research Institute, Mount Sinai Hospital, Toronto, ON Canada; 2 Department of Physiology, University of Toronto, Toronto, ON Canada; 3 Department of Obstetrics and Gynaecology, University of Toronto, Toronto, ON Canada; 4 Institute of Medical Science, University of Toronto, Toronto, ON Canada

**Keywords:** Stem-cell differentiation, Embryonic stem cells

## Abstract

In order to harness the potential of pluripotent stem cells, we need to understand how to differentiate them to our target cell types. Here, we developed a protocol to differentiate mouse embryonic stem cells (ESCs) to renal progenitors in a step-wise manner. Microarrays were used to track the transcriptional changes at each stage of differentiation and we observed that genes associated with metanephros, ureteric bud, and blood vessel development were significantly upregulated as the cells differentiated towards renal progenitors. Priming the ESCs and optimizing seeding cell density and growth factor concentrations helped improve differentiation efficiency. Organoids were used to determine the developmental potential of the renal progenitor cells. Aggregated renal progenitors gave rise to organoids consisting of LTL+/E-cadherin+ proximal tubules, cytokeratin+ ureteric bud-derived tubules, and extracellular matrix proteins secreted by the cells themselves. Over-expression of key kidney developmental genes, *Pax2*, *Six1*, *Eya1*, and *Hox11* paralogs, during differentiation did not improve differentiation efficiency. Altogether, we developed a protocol to differentiate mouse ESCs in a manner that recapitulates embryonic kidney development and showed that precise gene regulation is essential for proper differentiation to occur.

## Introduction

Pluripotent stem cells (PSCs), such as embryonic stem cells (ESCs) and induced PSCs (iPSCs), have the ability to self-renew and differentiate into all cells of all three germ layers, including renal progenitors. Renal progenitors, which include nephron progenitors, ureteric bud cells, and stromal progenitors, are multipotent stem cells capable of self-renewal and differentiation to all the specialized cell types in the adult kidney. These properties make renal progenitors a valuable cell source for therapeutic applications and regenerative medicine. Since the renal progenitor pool is exhausted shortly after birth, we propose to produce embryonic kidney-like cells in vitro by differentiating PSCs to multipotent renal progenitors. When cultured under conditions that mimic embryonic kidney development, PSCs can be differentiated to renal progenitors and coaxed to form kidney organoids, and these cells can be used in various applications, including cell-based therapy and nephrotoxicity screening. Here, we improved on existing mouse differentiation protocols by eliminating the use of embryoid bodies and developing a monolayer protocol that generates multiple renal progenitor cell types, including nephron progenitors, ureteric bud cells, and stromal progenitors.

The strategy to developing an in vitro differentiation method is to recapitulate embryonic kidney development. Mammalian kidney development begins with the formation of the pronephric duct at around E8.0 in mice, which extends caudally towards the hind limbs. As the nephric duct extends, it induces the formation of the mesonephros on the adjacent intermediate mesoderm at around E9.0 in mice. The metanephros starts to form at around E10.5 in mice when the nephric duct reaches a mass of cells called metanephric blastema. The nephric duct induces the metanephric blastema to become the metanephric mesenchyme, and, in turn, the metanephric mesenchyme induces the formation and outgrowth of the ureteric bud from the nephric duct. The cross-talk between the metanephric mesenchyme and ureteric bud is important for survival, migration, and proliferation of the two cell populations. The metanephric mesenchyme condenses to form pretubular aggregates, and the pretubular aggregates continue to undergo morphological changes and mesenchymal-to-epithelial transition to give rise to renal vesicles, comma-shaped bodies, S-shaped bodies, and eventually connect with the collecting duct and ureter to form a continuous tubular system.

From seminal research studies on the cellular and molecular basis of kidney induction and patterning, we know that activin A, bone morphogenetic proteins (Bmps), fibroblast growth factors (Fgfs), and Wnt molecules play important roles in inducing and promoting renal differentiation from primitive streak cells^1–4^. In addition to regulatory molecules, some key transcriptional factors and co-activators directly involved in inducing and maintaining renal progenitors include *Pax2*, *Six1*, *Eya1*, and *Hox11* paralogs (*Hoxa11*, *Hoxc11*, and *Hoxd11*). Knockout mouse studies show that defects in these genes result in metanephric mesenchyme apoptosis and lack of ureteric bud formation^5–8^. In humans, defects in these genes result in various renal abnormalities, including branchio-oto-renal syndrome, renal hypodysplasia, and renal-coloboma syndrome^9^.

There has been tremendous advancement in human PSC differentiation, but very little advancement in mouse PSC differentiation. Given the importance and power of transgenic rodent models in studying kidney development and disease, we recognize the need for a more reliable, reproducible, and a better characterized murine renal differentiation protocol. Currently, almost all mouse differentiation protocols use embryoid bodies as an intermediate step to generate renal progenitors^10–15^. Embryoid bodies are formed by aggregating and culturing PSCs as a cell aggregate, and factors such as embryoid body size and cell position within the embryoid body would affect differentiation. These protocols opt to use embryoid bodies as a method of differentiation, probably because mouse PSCs are notorious for being difficult to grow as a monolayer due to their “naive” pluripotency state as opposed to the “primed” pluripotency state of human PSCs, which are easier to grow as a monolayer. In addition, these studies did not differentiate ESCs to renal progenitors in a step-wise manner to recapitulate embryonic kidney development and often rely on transplanting the embryoid bodies into mice for maturation^10,11^. Lastly, only one progenitor population is generated in these protocols, while developmental studies demonstrate that the interactions between nephron progenitors, ureteric bud cells, and stromal progenitors are crucial for proper kidney development to occur.

Understanding these issues, we developed a protocol to direct the differentiation of mouse ESC, as a monolayer, to renal progenitors in a step-wise fashion. We incorporated a priming step in our differentiation protocol in order to generate a monolayer and prime the cells for mesoderm differentiation. Microarray analysis at each step of differentiation showed that ESCs were differentiating towards the kidney lineage. At the end of the 21-day protocol, gene expression and functional studies demonstrated that our protocol yields a mixture of renal progenitors, including nephron progenitors, ureteric bud cells, and stromal progenitors. When aggregated and cultured on an air–liquid interface, the renal progenitors formed kidney organoids containing LTL+/E-cadherin+ proximal tubule cells and cytokeratin+ ureteric bud-derived tubules, and all the tubules were supported by extracellular matrix proteins produced by the cells themselves. A kidney organoid made up of primarily tubules is useful for drug-induced toxicity studies since many nephrotoxic drugs target tubules, in particular proximal tubules. The inability to fully mature organoids in vitro is an ongoing issue in the field, and, at best, researchers are only able to generate human kidney organoids in vitro that are equivalent to second trimester fetal kidneys^16^. We cultured our mouse organoids for up to 3 weeks of in vitro, mirroring the full mouse gestation time, and found that they did not fully mature to express organic anionic transporter 1 and 3 (*Oat1*, *Oat3*) and organic cation transporter 2 (*Oct2*), which are drug transporters commonly found on mature proximal tubule cells. Since mirroring gestation time was not sufficient to yield mature adult organoids, it suggests that developmental cue(s) may be missing from our culture.

Lastly, we investigated whether we could improve differentiation at the genetic level by over-expressing *Pax2*, *Eya1*, *Six1*, *Hoxa11*, *Hoxc11*, and *Hoxd11* transgenes during ESC differentiation. With our system we found that over-expression of *Pax2*, *Eya1*, *Six1*, *Hoxa11*, *Hoxc11*, and *Hoxd11* inhibited differentiation, indicating that the precise regulation of gene expression is critical for proper development. Having a robust differentiation protocol will allow researchers to study kidney differentiation and organogenesis in vitro, and therefore allows researchers to gain a better understanding of these complex processes. This understanding will ultimately help develop tools for human kidney disease diagnosis, management, and treatment.

## Results

### Directed differentiation of mouse ESCs to renal progenitors

**Fig. 1:**
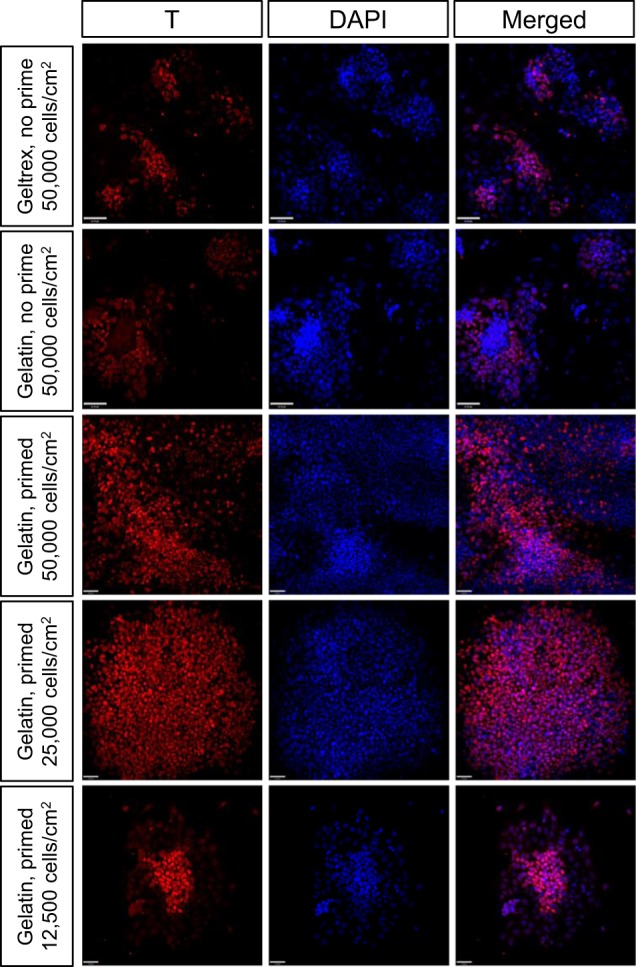
Mesoderm induction under different culture conditions. Immunocytochemistry on cells after mesoderm induction shows that T induction is affected by tissue culture plate coating, the absence/presence of priming induction, and initial seeding cell density. Scale bar = 40 µm.

To recapitulate kidney development in vitro, we developed a step-wise protocol to direct the differentiation of mouse ESCs to mesoderm, intermediate mesoderm, and renal progenitors. First, we empirically established the optimal conditions, including growth factor combinations, the substrate on which cells are cultured and differentiated, and the seeding cell density for mesoderm induction. Bmp4 and activin A are strong inducers of mesoderm cells, and activin A and Fgf2 have been shown to upregulate brachyury (T) expression^3^. Different concentrations, combinations, and culture durations of Bmp4 (20–30 ng/ml), activin A (10–30 ng/ml), and Fgf2 (10–50 ng/ml) were tested and a combination of 30 ng/ml Bmp4, 10 ng/ml activin A, and 12 ng/ml Fgf2 for 2 days was found optimal for mesoderm induction (Fig. 1).

The extracellular matrix plays a critical role in differentiation, and thus gelatin and Geltrex, which are commonly used in cell culture and differentiation, were tested. Gelatin is composed of mainly collagen, while Geltrex is a mixture of extracellular matrix proteins. We plated mouse ESCs at 50,000 cells/cm^2^ and differentiated them on gelatin-coated and Geltrex-coated tissue culture plates, and found that ESCs differentiated to T+ mesoderm more readily on gelatin-coated plates than Geltrex-coated plates (56.7 ± 6.4% vs. 22.3 ± 11.4% T+ cells, *p* < 0.05; Fig. 1).

Next, we explored whether adding a priming step in our differentiation protocol would improve differentiation efficiency. We hypothesized that the addition of a priming step consisting of Fgf2 and activin A to first prime the naive ESCs to epiblast stem cells (EpiSCs) would allow the cells to differentiate more readily to T+ mesoderm. In a study by Sudheer et al.^17^, they showed that culturing mouse ESCs in the presence of Fgf2 and activin A for 2 days was sufficient to convert ESCs to EpiSCs. Therefore, we plated mouse ESCs at 50,000 cells/cm^2^ and differentiated mouse ESCs to T+ mesoderm with and without a 2-day priming step. We found that the percent T+ cells in prime and no prime conditions were not statistically different from each other (prime: 49.8 ± 12.2% versus no prime: 56.7 ± 6.4%; Fig. 1). However, there were more T+ cells in the prime condition than the no prime condition (prime: 661/1366 T+ cells versus no prime: 175/303 T+ cells).

Finally, the impact of seeding cell density on mesoderm induction was assessed. Cell–cell contact is important for cell–cell communication and therefore affects cellular differentiation. Too few or too many cells could affect differentiation. We seeded ESCs at three cell densities at the start of differentiation (12,500, 25,000 or 50,000 cells/cm^2^) and found that ESCs seeded at 25,000 cells/cm^2^ yielded the most T+ cells after mesoderm induction (Fig. 1). When cells were seeded at 12,500, 25,000 and 50,000 cells/cm^2^, it yielded 37.3 ± 13.8%, 82.1 ± 17.0% and 49.8 ± 12.2% T+ cells, respectively. T induction was statistically higher (*p* < 0.05) when ESCs were seeded at 25,000 cells/cm^2^ compared to 12,500 or 50,000 cells/cm^2^. These optimized initial conditions were then used to establish the remaining differentiation protocol.

**Fig. 2:**
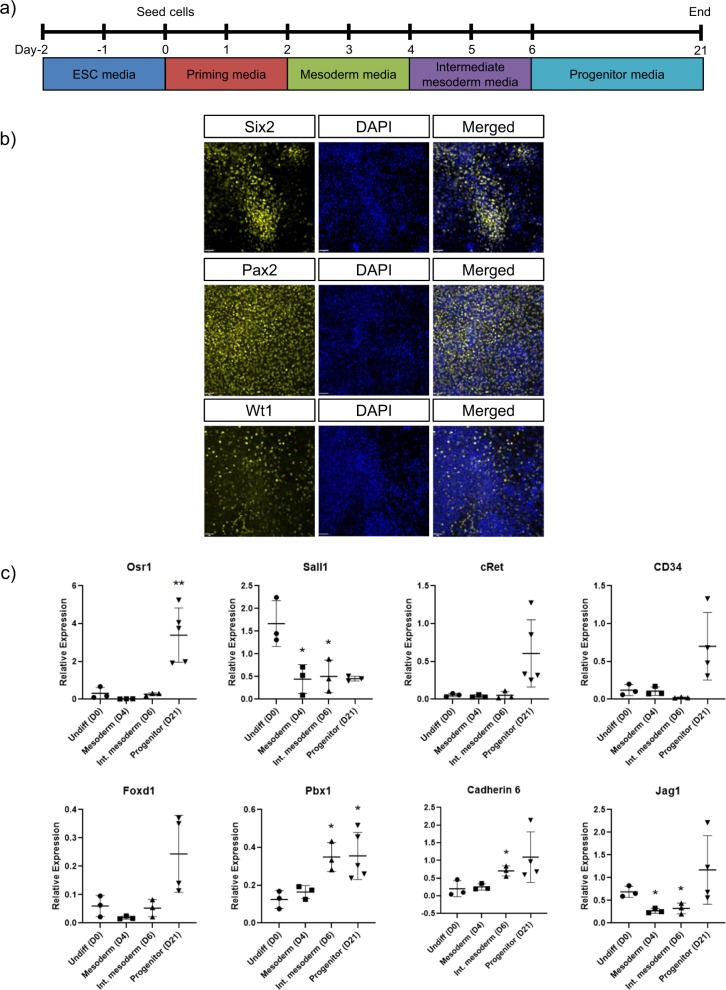
Renal progenitors derived from mouse embryonic stem cells (ESCs). **a** Schematic diagram of the renal differentiation timeline. **b** Immunocytochemistry reveals that the differentiated cells express Six2, Pax2, and Wt1 at the end of the 21-day differentiation protocol. Scale bar = 40 µm. **c** Quantitative PCR shows that the expression of renal progenitor markers changes as the embryonic stem cells (“Undiff”) differentiate to mesoderm, intermediate mesoderm, and progenitor. Expression is relative to E13.5 embryonic kidney. Asterisks (*) indicate that expression is significantly different (*p* ≤ 0.05 (*), *p* ≤ 0.01 (**)) from undifferentiated mouse embryonic stem cells, as determined by *t* test with Welch’s correction. *n* = 3–5. Error bars represent standard deviation.

Activin A, retinoic acid, ROCK inhibition, Fgf2, and Bmp7 are involved in intermediate mesoderm and renal progenitor induction^1,2,18–24^. Culturing T+ mesoderm in 10 µM Y27632 (ROCK inhibitor), 100 nM retinoic acid, and 30 ng/ml activin A for 2 days induced Pax2+ intermediate mesoderm formation. Next, culturing Pax2+ intermediate mesoderm cells in 150 ng/ml Bmp7 and 50 ng/ml Fgf2 for 15 days yielded 43.2 ± 8.7% Six2+, 16.7 ± 6.3% Wt1+, and 65.0 ± 5.3% Pax2+ progenitors (Fig. 2a, b). Six2, Wt1, and Pax2 are markers of renal progenitors and are expressed in E13.5 embryonic kidney cells, but not in undifferentiated mouse ESCs (Supplementary Fig. 1). Secondary antibody controls confirmed that secondary antibodies did not bind non-specifically to cells (Supplementary Fig. 2).

Using quantitative polymerase chain reaction (PCR), we showed that our differentiation protocol yields a mix of renal progenitors, including metanephric mesenchyme (*Osr1*, *Sall1*), nephric duct (*cRet*), endothelial progenitors (*CD34*), and stromal progenitors (*Foxd1*, *Pbx1*) (*n* = 3; Fig. 2c). We found that the expression of these markers was absent/low in undifferentiated ESC, but increased as the ESCs differentiated to mesoderm, intermediate mesoderm, and progenitors. After differentiation, the progenitor cells expressed their respective markers at levels that were similar to E13.5 mouse embryonic kidney cells. Interestingly, at the end of the 21-day differentiation protocol, some cells expressed *Cadherin 6* and *Jag1*, which indicated that some of the metanephric mesenchyme had differentiated further to renal vesicles. Finally, immunocytochemistry was used to reveal that pluripotency-associated marker Oct4 was absent in ESC-derived progenitors, and quantitative PCR was used to show that pluripotency-associated markers *Esrrb*, *Lefty2*, and *Lin28a* were significantly downregulated in progenitors compared to undifferentiated ESCs (Supplementary Fig. 3). This indicated that the differentiated renal progenitors are not pluripotent.

### Genome-wide gene expression analysis of cells during renal differentiation

**Fig. 3:**
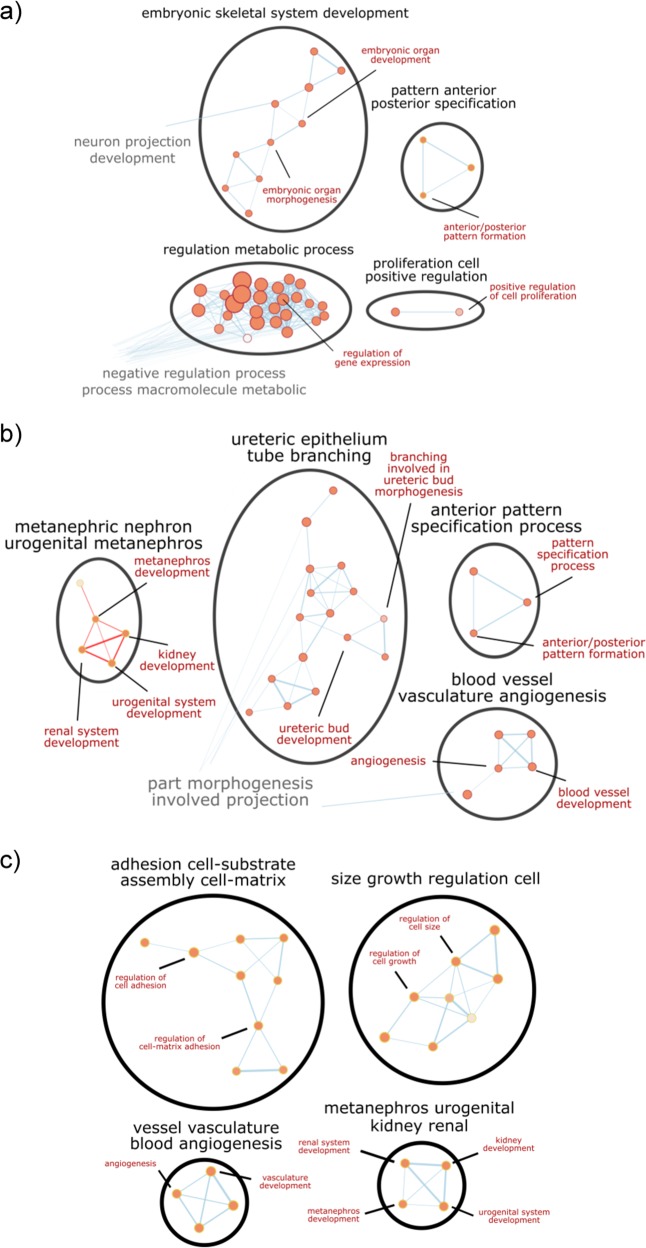
Network diagrams of selected gene ontology clusters enriched at each stage of differentiation. **a** Over-represented gene ontologies significantly enriched in mesoderm cells compared to undifferentiated mouse ESCs include embryonic organ development and morphogenesis, anterior/posterior pattern formation, and regulation of cell proliferation. **b** Over-represented gene ontologies significantly enriched in intermediate mesoderm cells compared to mesoderm cells include metanephros development, ureteric bud development, and vasculature development. **c** Over-represented gene ontologies significantly enriched in progenitor cells compared to intermediate mesoderm cells include kidney development, regulation of cell adhesion, regulation of cell–matrix adhesion, and vasculature development. FDR-corrected *p* < 0.05.

Principal component analysis (PCA) plots show that PC1 (43.2% variance) separates embryonic kidneys and ESC-derived progenitors from undifferentiated ESC, mesoderm, and intermediate mesoderm, and PC2 (20.5% variance) separates ESC-derived progenitors from embryonic kidneys, undifferentiated ESC, mesoderm, and intermediate mesoderm (Supplementary Fig. 4a). This shows that ESC-derived progenitors are more transcriptionally similar to embryonic kidneys than undifferentiated ESC, mesoderm, and intermediate mesoderm. Next, we used limma to determine significant differentially expressed genes between the different stages of differentiation, and we fed these lists into over-representation analysis to determine the enriched gene ontologies (GOs) associated with each stage of differentiation. Binary comparison of undifferentiated mouse ESCs to mesoderm cells revealed that 1941 genes were differentially expressed between them. Of the 1941 genes, 880 genes were significantly upregulated (false discovery rate (FDR)-corrected *p* value < 0.05) in mesoderm cells compared to undifferentiated ESC. Over-representation analysis of these genes revealed that GOs enriched in mesoderm cells included cell differentiation, development and morphogenesis, organ development and morphogenesis, and anterior/posterior pattern formation (Fig. 3a). These are all functions involved in gastrulation and the formation of mesoderm.

As the mesoderm cells differentiated to intermediate mesoderm cells, 1916 genes were differentially expressed with 881 genes significantly upregulated (FDR-corrected *p* value < 0.05) in intermediate mesoderm cells compared to mesoderm cells. Over-represented GOs enriched in intermediate mesoderm cells include metanephros development, epithelium development, ureteric bud development, and vasculature development (Fig. 3b). This supports the notion that our differentiation protocol yields nephron progenitors, ureteric bud cells, and endothelial progenitors.

Finally, the differentiation of intermediate mesoderm cells to renal progenitors resulted in 2842 differentially expressed genes, of which 958 genes were significantly upregulated (FDR-corrected *p* value < 0.05) in progenitors compared to intermediate mesoderm cells. The over-represented GOs enriched in progenitors include kidney development, regulation of cell adhesion, and regulation of cell–matrix adhesion (Fig. 3c). The sustained enrichment of kidney developmental genes indicated that the progenitor induction media promoted the continued differentiation of renal progenitors.

**Fig. 4:**
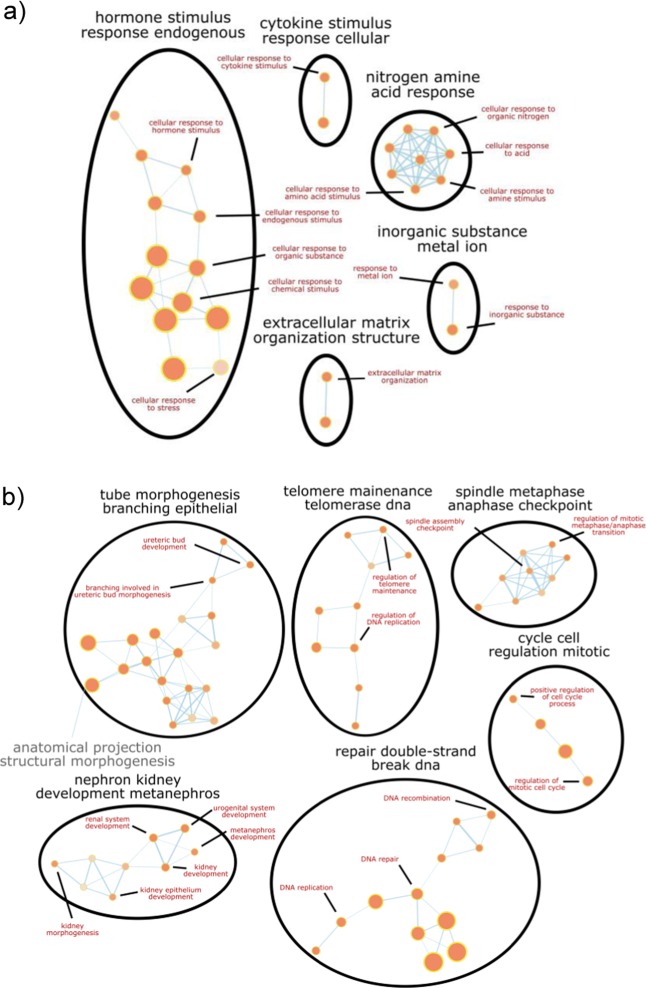
Network diagrams of selected gene ontology clusters enriched in ESC-derived renal progenitors compared to E12.5/13.5 mouse embryonic kidneys and vice versa. **a** Over-represented gene ontologies significantly enriched in progenitor cells compared to E12.5/13.5 embryonic kidneys include extracellular matrix organization and cellular response to stress, hormone, organic substance, and cytokine. **b** Over-represented gene ontologies significantly enriched in mouse embryonic kidneys compared to ESC-derived progenitors include kidney development, DNA replication, repair and recombination, and regulation of cell cycle. FDR-corrected *p* < 0.05.

**Fig. 5:**
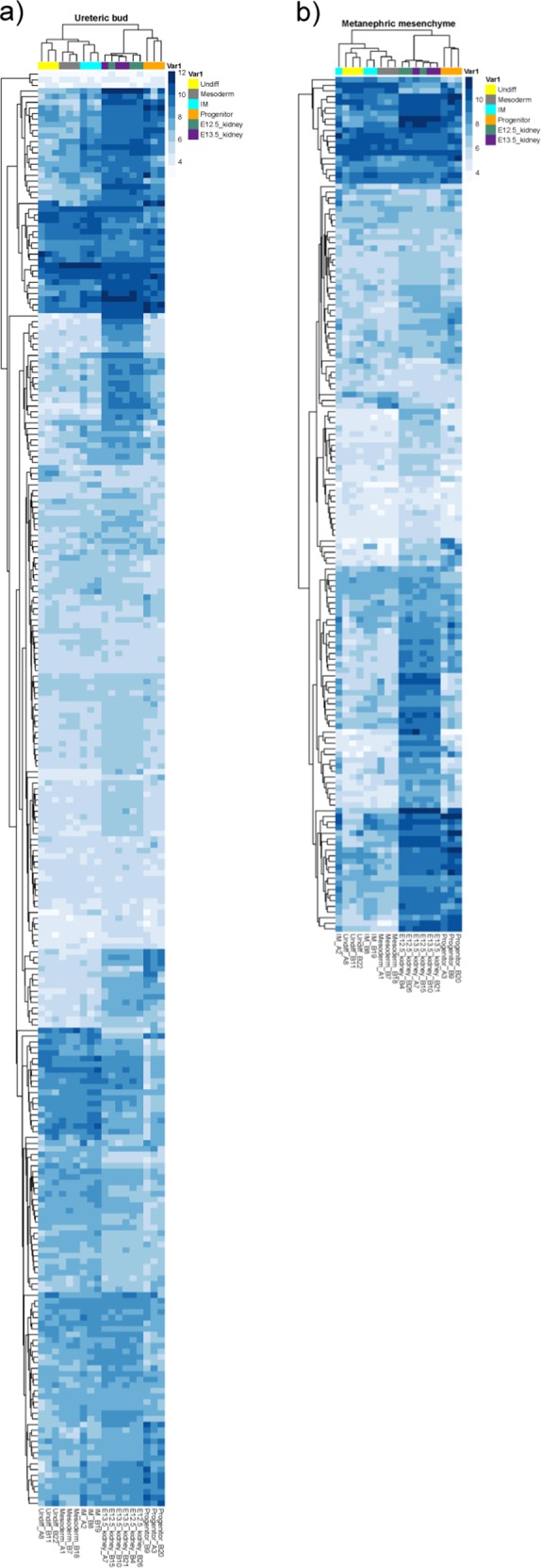
Heatmap depicting expression of metanephric mesenchyme- and ureteric bud-associated genes in undifferentiated mouse embryonic stem cells (ESCs), mesoderm cells, intermediate mesoderm cells, progenitors, and embryonic kidneys. The gene expression profiles of ESC-derived progenitors closely resemble E12.5/13.5 kidneys for **a** ureteric bud-associated genes and **b** metanephric mesenchyme-associated genes.

Binary comparison of ESC-derived renal progenitors and E12.5/E13.5 embryonic kidney would reveal genes differentially expressed between the samples and indicate what genes are lacking in each sample with respect to the other. Seven thousand and eight genes were differentially expressed between ESC-derived renal progenitors and embryonic kidneys. Of the 7808 genes, 3201 genes were significantly upregulated (FDR-corrected *p* value < 0.05) in ESC-derived progenitors compared to embryonic kidneys, and 4607 genes were significantly upregulated in embryonic kidneys compared to progenitors. Over-represented GOs enriched in ESC-derived progenitors include extracellular matrix organization, regulation of cell migration, and cellular response to stress, hormone, organic substance, and cytokine (Fig. 4a). On the other hand, GOs over-represented in embryonic kidneys include kidney development, nucleotide biosynthesis, and DNA replication, repair, and recombination (Fig. 4b). Importantly, focusing on 190 metanephric mesenchyme-associated genes and 298 ureteric bud-associated genes, the gene expression profiles of renal progenitors were more similar to E12.5/13.5 mouse embryonic kidneys, then undifferentiated ESC, mesoderm cells, and intermediate mesoderm cells (Fig. 5).

Altogether, our microarray data suggest that mouse ESCs are differentiated towards the renal lineage in our differentiation protocol, and that the expression of kidney developmental genes increase with each step of differentiation, resulting in enrichment in kidney-related genes and GOs.

### Renal progenitors give rise to kidney organoids when aggregated and cultured on an air–liquid interface

Mouse embryonic kidneys have the intrinsic ability to self-organize into renal structures when dissociated into single cells, aggregated, and cultured as a cell aggregate on an air–liquid interface. These structured cell aggregates are also referred to as organoids. We dissociated and cultured E13.5 mouse embryonic kidney cells on a porous filter and showed that they give rise to N-cadherin+ proximal tubules and cytokeratin 8+ ureteric bud-derived structures (Supplementary Fig. 5a). Similarly, when we dissociated our ESC-derived renal progenitors into single cells and cultured them on an air–liquid interface, they gave rise to the same structures as embryonic kidney cells (Supplementary Fig. 5b). No exogenous cells were added to the organoid culture so all the structures were derived from ESC-derived renal progenitors.

**Fig. 6:**
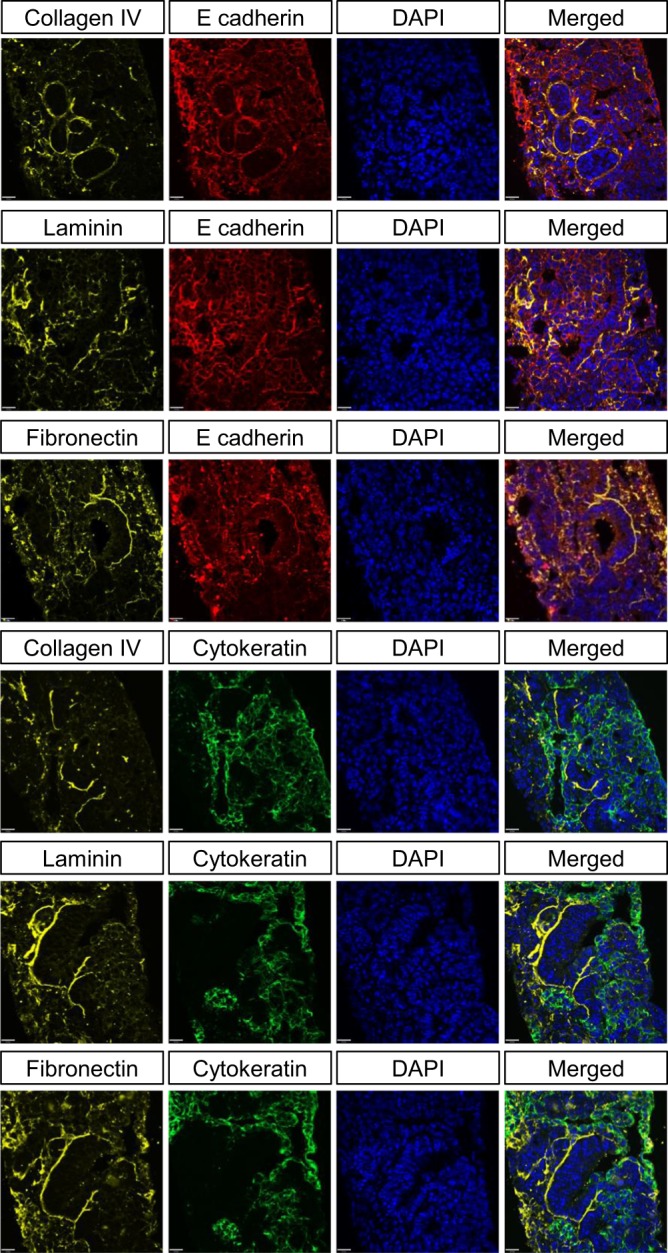
Expression of extracellular matrix proteins in kidney organoids containing E-cadherin+ and cytokeratin (pan)+ tubules. Immunohistochemistry of cross-sections of kidney organoids reveals that E-cadherin+ and cytokeratin (pan)+ tubules are surrounded by and supported by collagen IV, laminin, and fibronectin. Scale bar = 20 µm.

A closer look at cross-sections of our organoids revealed that the tubules, indicated by E-cadherin and cytokeratin (pan), were surrounded and supported by extracellular matrix proteins, collagen IV, laminin, and fibronectin (Fig. 6). Since no extracellular matrix proteins or scaffolds were used during the aggregation and culture of the organoids, all the observed extracellular matrix proteins were produced by the renal progenitors themselves. In addition, we found Pbx1+ stromal cells in the space between tubules, which is where they are found naturally (Supplementary Fig. 6). The expression of E-cadherin, cytokeratin, and aquaporin 2 suggests that we have tubules from both metanephric mesenchyme and ureteric bud origins (Supplementary Fig. 6). As a control, undifferentiated mouse ESCs were aggregated and cultured under the same conditions as kidney organoids to demonstrate that they do not give rise to the same tubule structures and extracellular matrix protein expression and organization as kidney organoids (Supplementary Fig. 7). Secondary antibody controls confirmed that our secondary antibodies did not bind non-specifically to cells (Supplementary Fig. 8).

**Fig. 7:**
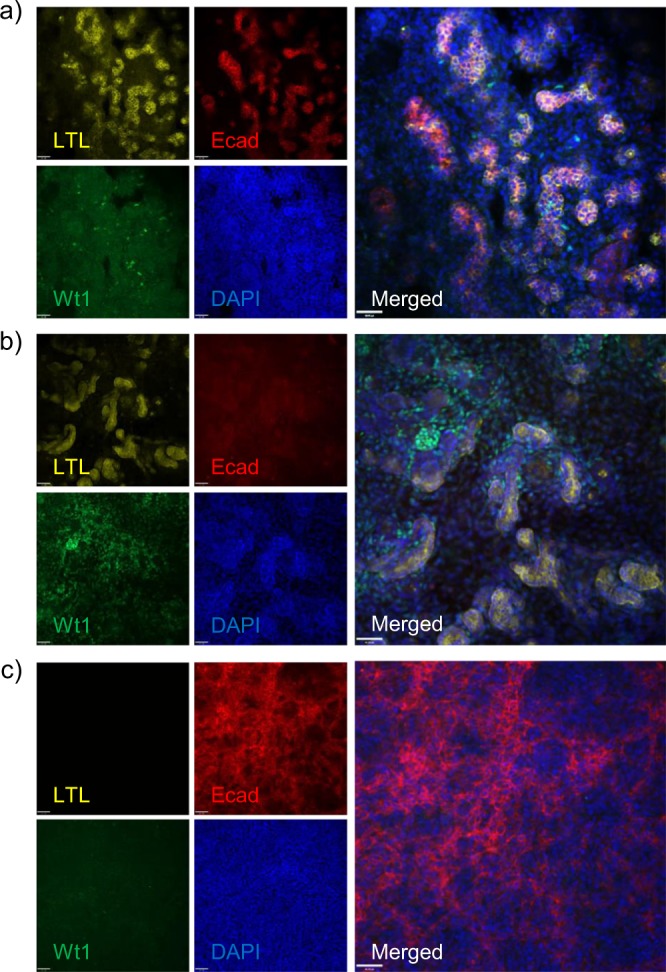
Expression of LTL, E-cadherin, and Wt1 in kidney organoids and cell aggregates. **a** Whole-mount staining of kidney organoid derived from embryonic stem cell (ESC)-derived renal progenitors shows the presence of LTL+/E-cadherin+ mature proximal tubules. **b** Embryonic kidney-derived organoids contain only LTL+ immature tubules. **c** Cell aggregates derived from ESCs do not contain LTL+ tubules. LTL lotus tetragonolobus lectin. Scale bar = 40 µm.

Next, we performed whole-mount staining on kidney organoids and cell aggregates made from mouse embryonic kidney, ESC-derived renal progenitors, and undifferentiated ESCs (Fig. 7). Kidney organoids made from ESC-derived renal progenitors gave rise to lotus tetragonolobus lectin (LTL)+/E-cadherin+ mature proximal tubules, but had few Wt1+ podocytes. On the other hand, kidney organoids made from E13.5 embryonic kidney cells had LTL+/E-cadherin immature proximal tubules, but many Wt1+ podocytes. This suggests that the organoids made from ESC-derived renal progenitors had more developed proximal tubules, but lacked podocytes compared to the organoids made from embryonic kidney cells. Our organoids are composed primarily of tubules because we added a Wnt agonist (CHIR99021) and Bmp7 to the organoid culture. Wnt and Bmp signaling play roles in patterning the kidney and determining the glomerulus-to-tubule ratio^25^. Similar to our results, work by Low et al.^26^ and Garreta et al.^16^ also showed that the addition of CHIR99021 results in a high tubule-to-glomerulus ratio. Lastly, as a control, cell aggregates from undifferentiated ESCs were made and they did not give rise to LTL+ tubules and Wt1+ podocytes, and the E-cadherin+ cells did not form any organized tubular structures. This shows that LTL+/E-cadherin+ proximal tubule cells do not spontaneously form in ESCs aggregates. Having a kidney organoid composed primarily of tubules is beneficial for studying tubule-specific development, disease, and toxicity.

### Gene expression analysis on kidney organoids cultured for up to 3 weeks in vitro

We cultured the organoids in vitro for 8 days (8D), 14 days (14D), or 21 days (21D) and used microarray to assess for changes in gene expression. PCA show that PC1 (59.1% variance) separates adult kidney from embryonic kidney, 8D organoid, 14D organoid, and 21D organoid. PC2 (17.7% variance) separates embryonic kidney from 8D organoid, 14D organoid, 21D organoid, and adult kidney (Supplementary Fig. 4b). This indicates that the organoids are more similar to mouse embryonic kidneys than adult kidneys, but has commonality with the adult kidney for some of the expressed genes.

**Fig. 8:**
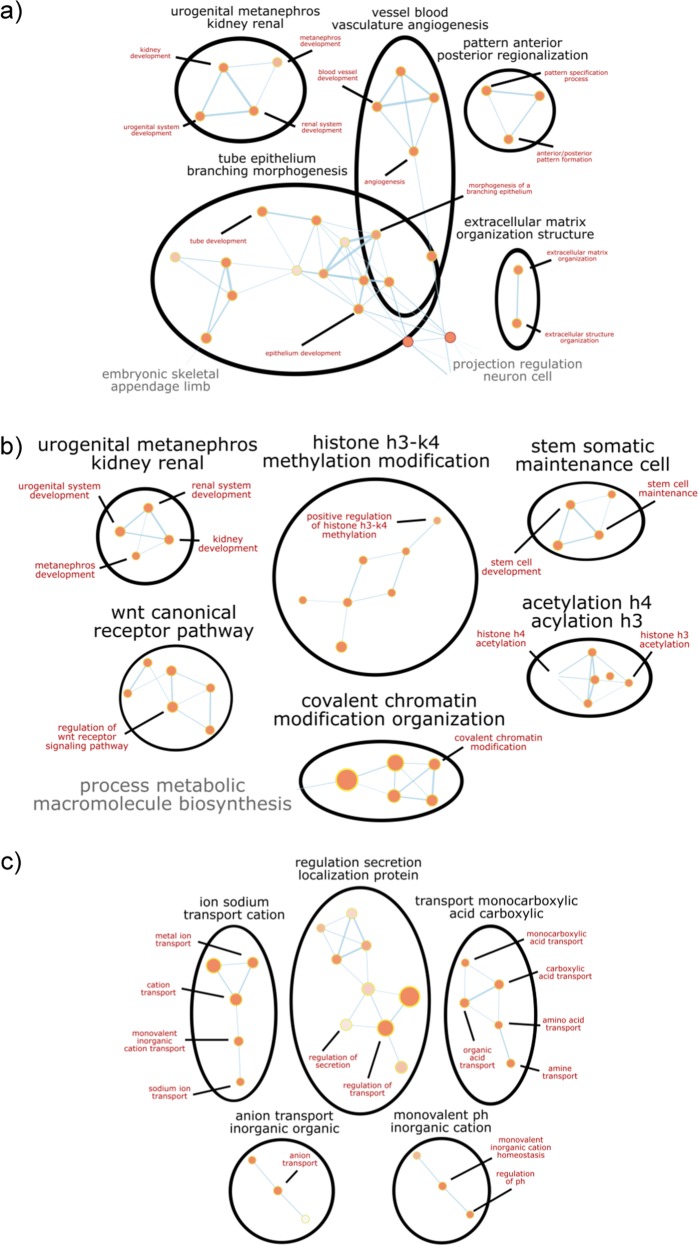
Network diagrams of selected gene ontology clusters enriched in 3D organoids and adult kidneys. **a** Over-represented gene ontologies enriched in 8D organoids compared to undifferentiated mouse embryonic stem cells (ESCs) include renal system development, extracellular matrix organization, blood vessel development, and anterior/posterior pattern formation. **b** Over-represented gene ontologies enriched in 8D organoids compared to adult kidneys include histone acetylation, histone methylation, metanephros development, and stem cell development, and maintenance. **c** Over-represented gene ontologies enriched in adult mouse kidneys compared to 8D organoids include regulation of pH, and anion, cation, carboxylic, and amine transport. FDR-corrected *p* < 0.05.

Binary comparison of undifferentiated mouse ESCs to 8D organoids revealed that there were 369 differentially expressed genes (FDR-corrected *p* value < 0.05). Of the 369 genes, 195 genes were significantly upregulated in 8D organoids. GOs enriched in 8D organoids include renal system development, extracellular matrix organization, and blood vessel development, indicating that the ESCs differentiated to kidney cells (Fig. 8a). Between 14D organoids and mouse ESC, and 21D organoids and mouse ESC, there were 5017 and 5806 differentially expressed genes (FDR-corrected *p* value < 0.05), respectively. 14D and 21D organoids had similar over-represented GO enrichments as 8D organoids (data not shown). There were no significant differentially expressed genes between 8D and 14D organoids, and 14D and 21D organoids, suggesting that the organoids did not mature further in vitro with extended culture. This is not surprising considering that the only proven method to mature kidney organoids is to transplant them into mice for extended in vivo culture.

Comparing 8D organoids and adult mouse kidneys, there were 9244 differentially expressed genes (FDR-corrected *p* value < 0.05). Of the 9244 genes, 4843 genes were significantly upregulated in 8D organoids and 4401 genes were significantly upregulated in adult kidneys. Examples of over-represented GOs significantly enriched in 8D organoids include regulation of cell division, histone acetylation, histone methylation, metanephros development, and stem cell development and maintenance (Fig. 8b). Some of the over-represented GOs enriched in adult kidneys compared to 8D organoids include regulation of pH, and anion, cation, carboxylic acid, and amine transport, suggesting that the cells in the organoids lack these functions (Fig. 8c). Together, these comparisons suggest that the cells in the organoids are immature and still in the development stage, and they do not exhibit the same functions as adult kidneys. Between 14D organoids and adult kidney, and 21D organoid and adult kidney, there were 10,384 and 9810 differentially expressed genes (FDR-corrected *p* value < 0.05), respectively. The over-represented GOs enriched in 14D and 21D organoids compared to adult kidneys were similar to the GOs enriched in 8D organoids (data not shown). These results are expected considering there were no significant differentially expressed genes between 8D and 14D organoids, and between 14D and 21D organoids.

**Fig. 9:**
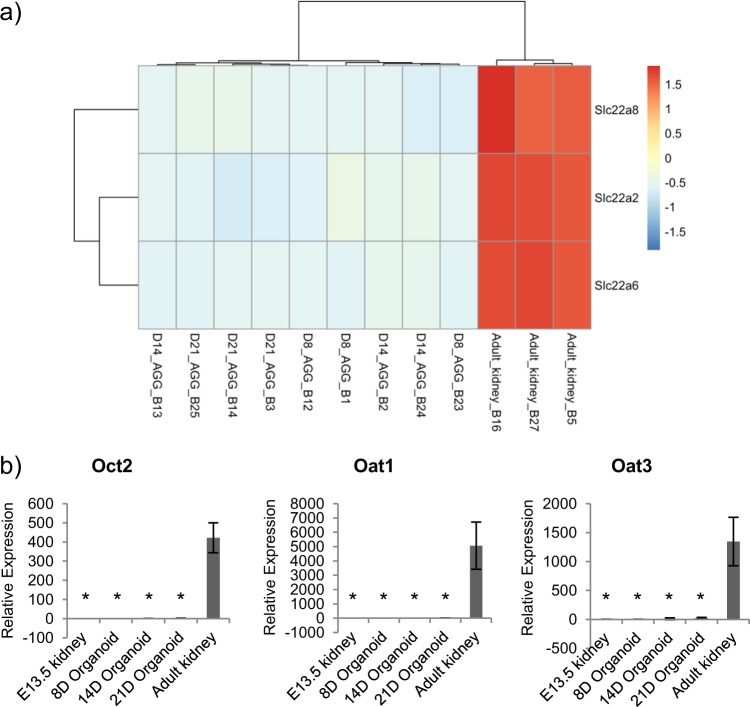
Expression of *Slc22a2* (*Oct2*), *Slc22a6* (*Oat1*), and *Slc22a8* (*Oat3*) expression in organoids and adult kidneys. **a** Heatmap of *Oct2*, *Oat1*, and *Oat3* expression in organoids and adult kidneys. **b** Quantitative PCR confirm that *Oct2*, *Oat1*, and *Oat3* are not expressed in organoids. Gene expression is relative to undifferentiated embryonic stem cells (ESCs). Asterisk (*) indicates that expression is significantly different (*p* < 0.05) from adult kidneys, as determined by *t* test. *n* = 3. Error bars represent standard deviation.

A characteristic of cellular maturity is the expression of drug transporter proteins on tubular cells. Cisplatin is an effective chemotherapy drug, but it is also nephrotoxic. Cisplatin-induced nephrotoxicity is mediated by Oct2 and Oat1 and Oat3. Oct2 mediates the uptake of cisplatin, while Oat1 and Oat3 mediate the uptake of mercapturic acid, which is a metabolite of cisplatin^27–29^. Our microarray data show that Oct2, Oat1, and Oat3 expression are significantly lower in 8D, 14D, and 21D organoids compared to adult mouse kidneys (Fig. 9a). This differential expression is also confirmed by quantitative PCR (Fig. 9b). Altogether, our gene expression data show that even with 3 weeks of culture, which mirrors mouse gestation time, there is still a large number of differentially expressed genes between organoids and adult kidneys, including drug transporters, indicating that our organoids are not at the same maturity level as adult kidneys. The inability to fully mature kidney organoids in vitro is an ongoing issue in the field.

### Over-expression of *Pax2*, *Eya1*, *Six1*, *Hoxa11*, *Hoxc11*, and *Hoxd11* during renal differentiation

*Pax2*, *Eya1*, *Six1*, and *Hox11* paralogs are transcription factors and co-activators involved in kidney development. We investigated whether the over-expression of *Six1*, *Pax2*, *Eya1*, *Hoxa11*, *Hoxc11*, and *Hoxd11* in mouse ESCs would improve renal differentiation efficiency and possibly maturation. To do this, we cloned the genes into a doxycycline (DOX)-inducible piggyBac (PB) transposon system using gateway cloning. A DOX-inducible system gives us temporal control of transgene expression.

**Fig. 10:**
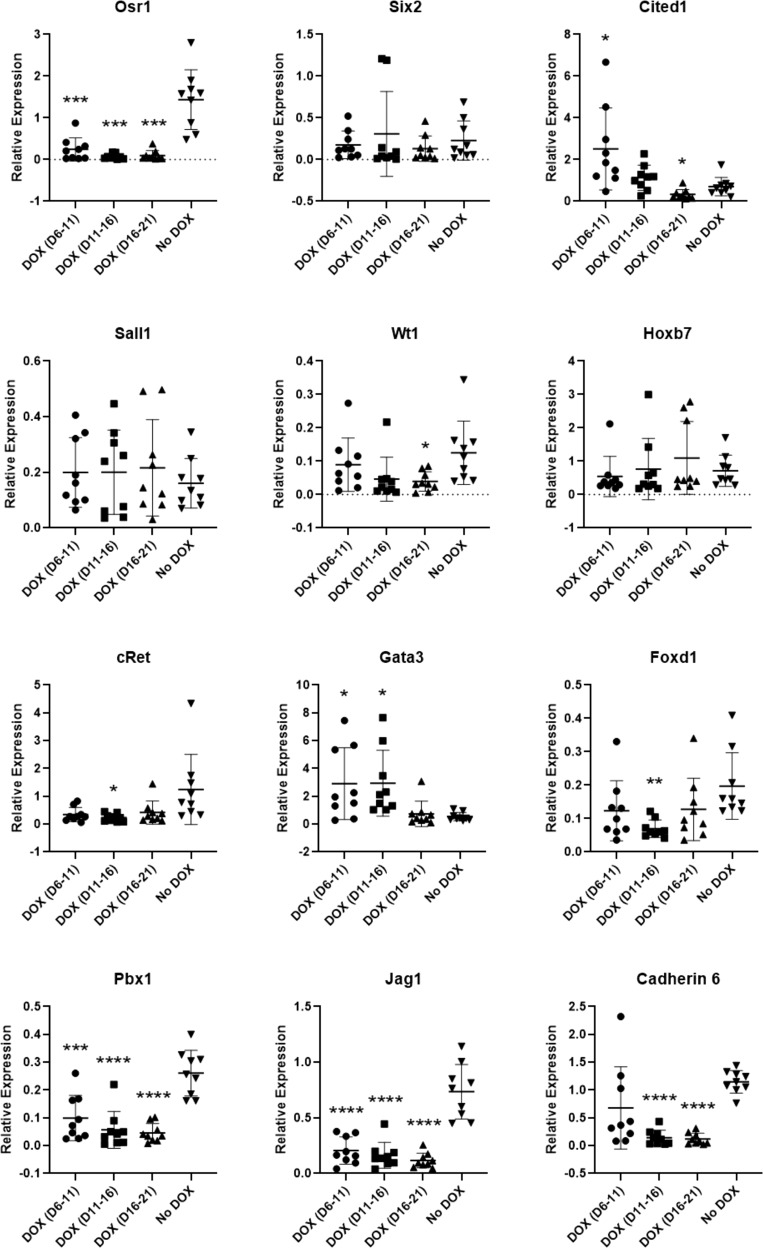
Progenitor marker expression of transgenic mouse embryonic stem cell (ESC) lines with doxycycline (DOX) added at different time points. Asterisks indicate that expression is significantly different (*p* ≤ 0.05 (*), *p* ≤ 0.01 (**), *p* ≤ 0.001 (***), *p* ≤ 0.0001 (****)) from the no DOX condition, as determined by *t* test with Welch’s correction. *n* = 3 cell lines, three replicates per line. Error bars represent standard deviation.

We introduced the transgenes into mouse ESCs using electroporation, and transgenic ESC clones were analyzed for the presence of the six transgenes (Supplementary Table I). Ten clones with all six transgenes were further characterized for transgene expression levels (Supplementary Table II). The three most DOX-responsive clones (clones 11, 19, and 23) were selected for renal differentiation. The transgenic ESCs were differentiated to renal progenitors using our established protocol with DOX added at different time points during differentiation. DOX was added at days 6–11, 11–16 or 16–21 of differentiation, which coincided with the progenitor induction stage of differentiation. When DOX was added from days 6 to 11, *Osr1*, *Jag1*, and *Pbx1* expression were significantly downregulated (*p* < 0.05) compared to the no DOX control group. On the other hand, *Gata3* and *Cited1* expression was significantly upregulated. When DOX was added from days 11 to 16, *Osr1*, *cRet*, *Foxd1*, *Pbx1*, *Jag1*, and *Cadherin 6* expression were significantly downregulated compared to the no DOX group, while *Gata3* continued to be significantly upregulated compared to the no DOX group. Lastly, when DOX was added from days 16 to 21, *Osr1*, *Cited1*, *Wt1*, *Pbx1*, *Jag1*, and *Cadherin 6* were significantly downregulated compared to the no DOX group (Fig. 10). Altogether, our data suggest that over-expression of *Six1*, *Pax2*, *Eya1*, *Hoxa11*, *Hoxc11*, and *Hoxd11* inhibits renal differentiation.

## Discussion

PSCs, which include ESCs and iPSCs, are capable of unlimited self-renewal and differentiation to cells of all three germ layers. These properties make them a powerful tool for studying and modeling embryogenesis, organogenesis, and cellular differentiation and regeneration. One major challenge faced by researchers is having a reliable and reproducible method to direct the differentiation of PSCs to the target cell type(s). By mimicking embryonic kidney development, we developed a step-wise protocol to direct the differentiation of mouse ESCs to mesoderm, intermediate mesoderm, and renal progenitors that resemble embryonic kidney cells. Briefly, our differentiation protocol consists of culturing ESCs as a monolayer in four induction media: priming, mesoderm, intermediate mesoderm, and progenitor. Each induction medium contains growth factors and small molecules tailored to the specific stage of kidney development. Unlike other published mouse differentiation protocols that utilizes an embryoid body step, our monolayer differentiation strategy gives us better control of differentiation trajectory and also yields metanephric mesenchyme, ureteric bud cells, and stromal cells.

In our protocol, mouse ESCs were differentiated on gelatin-coated plates at a seeding cell density of 25,000 cells/cm^2^. Gelatin is made from hydrolyzed collagen, and coating culture plates with gelatin is known to mediate cell attachment. We also differentiated cells on Geltrex-coated plates because we hypothesized that differentiating cells on Geltrex would improve differentiation since Geltrex is made from a mixture of extracellular matrix proteins and may provide important proteins for attachment and differentiation that are missing from gelatin. Contrary to our expectations, differentiating cells on Geltrex did not improve mesoderm induction, and this could be because mouse ESCs are grown and maintained on gelatin-coated plates prior to differentiation. In addition, Geltrex is a ready-to-use solution containing unknown growth factors, albeit at a low concentration, but they may be present at levels that interfere with mesoderm differentiation.

We and others have highlighted the importance of cell seeding density in defining cell fate and differentiation efficiency^30–33^. Cell density influences cell–cell interaction and paracrine signaling between cells, and ultimately influences cellular differentiation. Here, we showed that mouse ESCs behaved differently when seeded at low, intermediate, and high cell density with the intermediate cell density yielding the most T+ cells. It is important to note that the optimal seeding cell density may vary between cell lines and should be optimized for each cell line.

Mouse ESCs are derived from the inner cell mass of E3.5 blastocysts and give rise to the visceral and parietal yolk sacs and the epiblast. EpiSCs give rise to cells of all three germ layers and can be found in E4.5 blastocysts. Although ESCs and EpiSCs are both pluripotent in nature, there are fundamental differences between the two cell types^34^. Morphologically, ESC colonies appear as tight, compact domes, while EpiSC colonies are flat. ESC maintenance is dependent on leukemia inhibitory factor (LIF), while EpiSCs require activin A and Fgf2. Prior to mesoderm induction, we included a priming step in our differentiation protocol to convert ESCs to EpiSCs because we hypothesized that priming would improve mesoderm differentiation efficiency^17,35^. Not only would a priming step prime ESCs for differentiation to mesoderm, but it would also flatten the cells into a flat monolayer, which allows for better control of differentiation trajectory. Indeed, we found that the differentiation protocol yields more T+ cells when a priming step was included.

We developed our differentiation protocol using the monolayer method because it has advantages over the embryoid body method. Monolayer culture allows for equal distribution of nutrients and signaling molecules among the cells. Embryoid bodies can vary in size and it is difficult to have consistently uniform embryoid bodies. We reasoned that size inconsistency and heterogeneity could lead to variations in the differentiated population. More importantly, we showed that ESCs differentiated as a monolayer can give rise to renal progenitors that could be expanded in culture and still retain the expression of progenitor markers Six2, Pax2, and Wt1 (Supplementary Figs 9–11).

The step-wise structure of our differentiation protocol allowed us to examine genome-wide transcriptional changes at each step of differentiation. We found that the relative expression of kidney developmental genes increased with each step of differentiation, which resulted in enrichment for kidney-related GOs, including metanephros and ureteric bud development. This confirms that, unlike other mouse differentiation protocols, our protocol gives rise to both metanephric mesenchyme and ureteric bud progenitors. Binary comparison of ESC-derived progenitors and embryonic kidneys revealed that there were some fundamental differences between them. These differences lie in the level of kidney developmental gene expression, the extent of extracellular matrix organization and remodeling, and the cellular response to stimuli.

Stem cell organoids, or mini organs, are collections of stem/progenitors cells that self-organize to form tissue-specific structures through processes that resemble in vivo tissue maturation, namely, cell sorting and spatially restricted lineage commitment^36^. Organoids were first made by dissociating and re-aggregating embryonic tissue, but using the same rationale, tissue-specific stem/progenitor cells differentiated from PSCs could also be coaxed to form organoids. Since PSCs can be genetically engineered to harbor disease-specific mutations or they can be made from patient cells, PSC-derived organoids are ideal models to study organogenesis, disease initiation and progression, and drug-induced nephrotoxicity in vitro.

When the ESC-derived renal progenitors were aggregated and cultured on an air–liquid interface, the progenitors gave rise to metanephric mesenchyme-derived proximal tubules and ureteric bud-derived cytokeratin+ tubules, and the tubules were supported by extracellular matrix proteins, collagen IV, laminin, and fibronectin, which were secreted by the cells themselves. The extracellular matrix not only provides structural support but it also serves as a reservoir for secreted growth factors. The presence of all three major extracellular matrix components is important for normal tissue development. No study has looked at the extracellular matrix composition of mouse organoids, and in terms of human organoids, groups have only surveyed for the presence of laminin. Here, we demonstrate that our mouse organoids contain collagen IV, laminin, and fibronectin.

We used microarray to study the transcriptional differences between ESC-derived organoids and undifferentiated ESCs, and between ESC-derived organoids and adult mouse kidneys. When compared to undifferentiated ESCs, genes associated with renal system development, blood vessel development, and extracellular matrix organization were significantly upregulated in the kidney organoids, as expected. We cultured our kidney organoids for up to 3 weeks in vitro and found that at the transcriptional level, there were no significant differences between 8D and 14D organoids, and 14D and 21D organoids. Even though our organoids contained proximal tubules that co-expressed LTL and E-cadherin, indicating maturity, we found that there were >9000 significant differentially expressed genes between organoids and adult kidneys, including drug transporter genes *Oct2*, *Oat1*, and *Oat3*. This suggests that our organoids, even with 3 weeks of culture that mirrored mouse gestation, are not at the same maturity level as adult kidneys. Our results are in line with other groups’ results demonstrating that kidney organoids are difficult to mature in vitro and organoids often have to be transplanted into immunocompromised mice for long-term maturation^37–40^. With current technology, researchers are only able to generate human kidney organoids in vitro that are equivalent to second trimester fetal kidneys^16^.

It is speculated that it is difficult to mature organoids in vitro because culture conditions lack signaling molecules crucial for maturation and/or the length of in vitro culture has to mimic gestation time. Since mouse gestation is only 21 days, our mouse differentiation system allowed us to test whether mirroring gestation time enables kidney organoids to fully mature in vitro. In our system, we differentiated mouse ESCs to renal progenitors in 21 days, aggregated them to form kidney organoids and then cultured the kidney organoids for up to 3 weeks in vitro. Our culture time exceeds the length of mouse gestation, but our organoids did not fully mature. Therefore, it is more likely that our culture system is missing signaling molecules that are important for kidney maturation. Other groups have also shown that extended static in vitro culture of organoids does not improve organoid maturation and may be detrimental to the organoids^37,40^. Flow and shear stress have been implicated in cellular and tissue maturation, and considering that there is no blood flow in our organoids in vitro, this may be one of the limiting factors. Indeed, Homan et al.^41^ showed that culturing kidney organoids under flow on microfluidic chips helps mature organoids.

Lastly, we described a system in which we can study the effects of gene over-expression on kidney differentiation. Although the effects of gene knockout or knockdown on kidney development are well studied, the effects of gene over-expression are not. We investigated whether over-expression of *Pax2*, *Eya1*, *Six1*, and the *Hox11* paralogs could improve kidney differentiation. These genes form a gene regulatory network that ultimately activates and regulates Six2 expression and the progenitor cell population. Mutations in these genes result in kidney malformations or agenesis^5–8^. Given the importance of this gene expression network in kidney development, we hypothesized that over-expressing these genes would improve differentiation of ESCs to renal progenitors. Over-expressing six factors at once is not uncommon in the field of direct reprogramming or direct conversion of one cell type to another. For instance, Hendry et al.^42^ over-expressed six factors at once (SIX1, SIX2, OSR1, EYA1, HOXA11, and SNAI2) to convert adult proximal tubule cells to nephron progenitors. In another example, Kaminski et al.^43^ over-expressed four factors (Emx2, Hnf1b, Hnf4a, and Pax8) at the same time to convert fibroblast to renal tubular epithelial cells. In the most famous example of all, Takahashi and Yamanaka^44^ initially over-expressed 24 genes at once to convert mouse fibroblasts to iPSCs before they performed an elimination experiment to identify the four indispensable genes.

Using quantitative PCR as a measure of differentiation efficiency, we assessed whether over-expression of these six genes during progenitor induction would improve differentiation efficiency. Contrary to our hypothesis, over-expression of *Pax2*, *Eya1*, *Six1*, and *Hox11* paralogs inhibited differentiation as indicated by the marked reduction in the expression of progenitor markers, and since this did not yield renal progenitors, additional analysis such as cell aggregation and morphological assessments were not performed. We observed a marked reduction in progenitor marker expression because the relative expression levels and the timing of the six transgenes over-expression may be inappropriate for proper deployment of the kidney-specific gene expression network and/or the high doses of the six transgenes could be detrimental to the cell. In our system, it is difficult to control the relative expression levels of transgenes since we made the transgenic mouse ESC lines using the PB transposon system that inserts inverse terminal repeat-flanked transgenes into TTAA DNA sequences and so we have no control over the number of copies of transgenes inserted into the genome. Moreover, our system does not allow for sequential expression of the transgenes since all the transgenes are under the control of a DOX-inducible promoter. The next steps would be to investigate whether sequential expression of the six genes would improve differentiation.

In summary, we described in depth a strategy to differentiate mouse ESCs to renal progenitors and kidney organoids, and how this system can be modified to study the effects of gene over-expression on kidney differentiation. Our results highlight the importance of monolayer culture, seeding cell density, extracellular matrix, growth factor, and gene dose on kidney differentiation. In order to fully harness the potential of kidney organoids in modeling advanced diseases and nephrotoxicity, future studies need to focus on developing strategies to mature and pattern organoids to resemble adult kidneys.

## Methods

### Ethics statement

All experiments were approved by and performed at the Lunenfeld Tanenbaum Research Institute at Mount Sinai Hospital in Toronto, Canada. Only mouse cell lines were used in the experiments. No animals or human subjects/tissues were used.

### Maintaining mouse ESCs

Mouse ESC line 1B10 was maintained on mitomycin C-treated mouse embryonic fibroblasts (MEFs) and cultured in mouse ESC media (Dulbecco’s modified Eagle’s medium (DMEM) supplemented with 15% fetal bovine serum (FBS), 1000 U/ml LIF, 50 U/ml penicillin, 50 µg/ml streptomycin, 2 mM GlutaMAX, 0.1 mM non-essential amino acids (NEAA), 1 mM sodium pyruvate, and 0.1 mM 2-mercaptoethanol). Cells were kept in 5% CO_2_ and at 37 °C. The medium was changed every other day.

### Directed differentiation of mouse ESCs to renal progenitors

Prior to differentiation, mouse ESCs were cultured until they were 70–80% confluent. Confluent ESCs were dissociated into single cells with TrypLE Express (Invitrogen) and passed through a 70 µm cell strainer to obtain single cells. Cells were centrifuged at 200 × *g*, 10 °C for 5 min. The supernatant was discarded and the cell pellet was resuspended in Priming Media (DMEM/F12 containing 1× N2 supplement, 1× B27 supplement, 0.1 mM NEAA, 2 mM GlutaMAX, 50 U/ml penicillin, 50 µg/ml streptomycin, 12 ng/ml Fgf2, and 20 ng/ml activin A). Cells were plated onto 0.1% gelatin-coated plates at a seeding cell density of 25,000 cells/cm^2^. Cells were cultured in priming media for 2 days before the cells were washed once with phosphate-buffered saline (PBS) and cultured in mesoderm induction media (DMEM/F12 containing 4% knockout serum replacement (KSR), 0.1 mM NEAA, 2 mM GlutaMAX, 50 U/ml penicillin, 50 µg/ml streptomycin, 1 mM sodium pyruvate, 0.015% w/v sodium bicarbonate, 0.1 mM 2-mercaptoethanol, 30 ng/ml Bmp4, 10 ng/ml activin A, and 12 ng/ml Fgf2) for 2 days. After mesoderm induction, cells were washed once with PBS and cultured in an intermediate mesoderm induction media modified from Sambi et al.^24^ (DMEM/F12 containing 4% KSR, 50 U/ml penicillin, 50 µg/ml streptomycin, 10 µM Y27632, 100 nM retinoic acid, and 30 ng/ml activin A) for 2 days^24^. Lastly, cells were washed and cultured in progenitor induction media adapted from Kang and Han^45^ (RPMI-1640 containing 1× B27 supplement, 2 mM GlutaMAX, 50 U/ml penicillin, 50 µg/ml streptomycin, 150 ng/ml Bmp7, and 50 ng/ml Fgf2) for 15 days^45^. Cells were not passaged during the 21-day differentiation period.

### Quantitative PCR

RNA was isolated using the RNeasy Mini Kit (Qiagen), and complementary DNA (cDNA) was synthesized using Superscript II Reverse Transcriptase (Thermo Fisher) and amplified using the SensiFAST SYBR No ROX Kit (Bioline) under the following conditions: 95 °C for 15 s, followed by 40 cycles of 95 °C for 5 s, and 60 °C for 30 s. The primers used were as follows:

Osr1 (F: TACTCTTTCCTTCAGGCAGTGA, R: GATCGAGGCAAGTGCATGG),

Six2 (F: CACCTCCACAAGAATGAAAGCG, R: CTCCGCCTCGATGTAGTGC),

Cited1 (F: AACCTTGGAGTGAAGGATCGC, R: GTAGGAGAGCCTATTGGAGATGT),

Sall1 (F: CTCAACATTTCCAATCCGACCC, R: GGCATCCTTGCTCTTAGTGGG),

Wt1 (F: GAGAGCCAGCCTACCATCC, R: GGGTCCTCGTGTTTGAAGGAA),

Hoxb7 (F: AAGTTCGGTTTTCGCTCCAGG, R: ACACCCCGGAGAGGTTCTG),

Gata3 (F: CTCGGCCATTCGTACATGGAA, R: GGATACCTCTGCACCGTAGC),

cRet (F: GCGTCAGGGAGATGGTAAAG, R: CATCAGGGAAACAGTTGCAG),

Foxd1 (F: CGCTAAGAATCCGCTGGTGAAG, R: GGATCTTGACGAAGCAGTCGTT),

Pbx1 (F: CAGCGGGTTCTTCCAGTTCTT, R: CGAGTCCGTCACTGTATCCTC),

CD34 (F: AAGGCTGGGTGAAGACCCTTA, R: TGAATGGCCGTTTCTGGAAGT),

Jag1 (F: CCTCGGGTCAGTTTGAGCTG, R: CCTTGAGGCACACTTTGAAGTA),

Cadherin 6 (F: CAGCCCTACCCAACTTTCTCA, R: GAACGGCTCAGCTCATTCC),

β-Actin (F: GGCTGTATTCCCCTCCATCG, R: CCAGTTGGTAACAATGCCATGT),

Esrrb (F: GCACCTGGGCTCTAGTTGC, R: TACAGTCCTCGTAGCTCTTGC),

Lefty2 (F: CAGCCAGAATTTTCGAGAGGT, R: CAGTGCGATTGGAGCCATC),

Lin28a (F: GGCATCTGTAAGTGGTTCAACG, R: CCCTCCTTGAGGCTTCGGA),

Oct2 (F: TTGTTGGGCTGGGCTATCG, R: CCAGTTGGGAAGGGCATAGG),

Oat1 (F: CTGATGGCTTCCCACAACAC, R: GTCCTTGCTTGTCCAGGGG), and

Oat3 (F: ATGACCTTCTCCGAGATTCTGG, R: GTGGTTGGCTATTCCGAGGAT).

Quantitative PCR data was acquired and analyzed using the Bio-Rad CFX system. Expression data is displayed as the average of 3–5 replicates with standard deviation. Statistical significance between sample groups was calculated using unpaired *t* test (with Welch’s correction) on GraphPad Prism 8 and *p* values < 0.05 (two tailed) were considered significant.

### Microarray

RNA was isolated using the RNeasy Mini Kit (Qiagen) and RNA quality was assessed using the Agilent Bioanalyzer. Only RNA with a RNA integrity number of >7.0 were used for microarray. Samples were hybridized to Affymetrix Mouse Gene 2.0 ST array chips by a trained personnel at The Center for Applied Genomics (Toronto, Canada). Raw CEL files were read and normalized in R using the oligo and limma Bioconductor packages^46–48^. PCA plots were generated using ggplot2^49^. The limma package in R Bioconductor was used to determine significant differentially expressed genes for each comparison (FDR-corrected *p* value < 0.05). Gene set enrichment analysis (GSEA) was used for binary comparisons with <500 significant differentially expressed genes. GSEA was conducted using the R Bioconductor topGO package using the EMAPA database of mouse developmental anatomy ontology (http://www.obofoundry.org/ontology/emapa.html)^50^. To narrow down the ontologies related to adult kidneys, we did a binary comparison between ESC and adult mouse kidneys to create a subset of ontologies highly enriched in their respective tissues. The subset of ontologies enriched to adult mouse kidneys was applied to experimental samples using topGO to determine if genes contributing to the ontologies are significantly enriched. Statistical significance is defined as having a FDR-corrected *p* value < 0.05.

Over-representation analysis was used for binary comparisons with >500 significant differentially expressed genes. For over-representation analysis, the gene lists were entered into BiNGO on Cytoscape to conduct over-representation analysis for GO Biological Processes (Benjamin and Hochberg FDR-corrected *p* value < 0.05, ontologies downloaded from www.geneontology.com on August 2018)^51,52^. The output was then exported and replotted using the Enrichment Map plugin for Cytoscape with the Node Cutoff set as 0.0001 and Edge Cutoff set as 0.7^53^. AutoAnnotate and WordCloud plugins were used to generate clusters of terms using Community Cluster (GLay) cluster algorithm and the label algorithm using four of the biggest keywords, respectively^54,55^. Only selected GOs are shown in figures.

### Immunocytochemistry

The medium was removed and cells were washed twice in PBS. Cells were then fixed in 10% neutral-buffered formalin (NBF) for 20 min at room temperature (RT). Fixed cells were washed with PBS containing 0.1% Triton X-100, and incubated with blocking buffer (PBS containing 0.1% Triton X-100 and 10% FBS) for 30 min at RT. After blocking, primary antibodies (diluted 1:200) were added and incubated with the cells overnight at 4 °C. The next day, cells were washed 4× for 10 min with PBS containing 0.1% Triton X-100 prior to incubating with secondary antibodies (diluted 1:500) for 30 min in the dark and at RT. Finally, cells were washed 5 × 10 min with PBS containing 0.1% Triton X-100, stained with DAPI (4′,6-diamidino-2-phenylindole), and stored in PBS containing 50% glycerol, in the dark at 4 °C until imaging. Secondary antibody controls in which only the secondary antibodies were added were performed to confirm the specificity of the secondary antibody. The primary antibodies used were as follows: T (Santa Cruz, SC-17743), Six2 (ProteinTech, 11562-1-AP), Wt1 (Abcam, AB89901), Pax2 (Covance, PRB276P), and Oct4 (Santa Cruz, SC8628). The secondary antibodies used were as follows: anti-goat IgG 594 and anti-rabbit IgG Cy5.

### Microscopy

All images were taken on the Quorum WaveFX Spinning Disc Confocal System equipped with CSU X1 Confocal Scanner Unit (Yokogawa), ImagEM EM-CDD Camera (Hamamatsu), and a DMI6000 B Fully Automated Inverted Research Microscope (Leica). Images were acquired and processed on the Volocity Software Version 6.3.0 (Improvision/PerkinElmer).

### Quantifying differentiation efficiency

Cells were immunostained and imaged on the Quorum WaveFX Spinning Disc Confocal System. Cells from three to seven fields were counted using ImageJ. Specifically, an intensity threshold was selected for positive cells, and all objects that were above the threshold were quantified by the program. Differentiation efficiency was expressed as the number of positively stained cells divided by the number of DAPI+ cells, and with the standard deviation.

### DOX-inducible *Pax2*, *Eya1*, *Six1*, *Hoxa11*, *Hoxc11*, and *Hoxd11* expression vectors

Gene vectors for *Pax2* (V102758-1), *Eya1* (V71070-1), *Six1* (V77822-1), *Hoxa11* (V99883-1), *Hoxc11* (V115823-1), and *Hoxd11* (V118513-1) were purchased from Open Freezer (www.openfreezer.org). The genes were then amplified using PCR with their respective attB primers: *Pax2* (attB1: GGGGACAAGTTTGTACAAAAAAGCAGGCTACCATGGATATGCACTGCAAAGCAGA, attB2: GGGGACCACTTTGTACAAGAAAGCTGGGTCTAGTGGCGGTCATAGGCAG); *Eya1* (attB1: GGGGACAAGTTTGTACAAAAAAGCAGGCTACCATGGAAATGCAGGATCTAACCAG, attB2: GGGGACCACTTTGTACAAGAAAGCTGGGTTTACAGGTACTCTAATTCCA); *Six1* (attB1: GGGGACAAGTTTGTACAAAAAAGCAGGCTACCATGTCGATGCTGCCGTCGTTTGG, attB2: GGGGACCACTTTGTACAAGAAAGCTGGGTTTAGGAACCCAAGTCCACCA); *Hoxa11* (attB1: GGGGACAAGTTTGTACAAAAAAGCAGGCTACCATGATGGATTTTGATGAGCGTGG, attB2: GGGGACCACTTTGTACAAGAAAGCTGGGTTTAGAGAAGTGGATTAGCTG); *Hoxc11* (attB1: GGGGACAAGTTTGTACAAAAAAGCAGGCTACCATGTTTAACTCGGTCAACCTGGG, attB2: GGGGACCACTTTGTACAAGAAAGCTGGGTTTACAGCAGTGGATTTCCTG); and *Hoxd11* (attB1: GGGGACAAGTTTGTACAAAAAAGCAGGCTACCATGAACGACTTTGACGAGTGCGG, attB2: GGGGACCACTTTGTACAAGAAAGCTGGGTTCAAAATAAGGGGTTTCCAG). The genes were amplified using PrimeSTAR HS (Takara, R040A) and under the following conditions: 30 cycles of 98 °C for 10 s, 58 °C for 5 s, and 72 °C for 1 min. The correct size of PCR products were excised from agarose gels using the QIAquick Gel Extraction Kit (Qiagen, 28704). The PCR products were then inserted into pDONR vectors to generate entry vectors using the Gateway BP Clonase II Enzyme Mix (Invitrogen, 11789-020). The entry vectors were transfected into DH5α-competent bacteria, amplified, and purified. The gene of interest was then cloned into the destination vectors using Gateway LR Clonase II Enzyme Mix (Invitrogen, 11791-020). The destination vector (courtesy of Dr. Andras Nagy) has a DOX-inducible promoter, a mCherry reporter, and a puromycin-selectable marker. The expression vectors were sequenced and gene inserts were 97%+ identical to their respective native gene sequences.

### Generating transgenic mouse ESC lines

ESCs were cultured under standard conditions and grown until 70–80% confluent. Confluent ESCs were collected, dissociated into single cells, and electroporated using the Neon Electroporation System (Invitrogen). Cells were electroporated under the following parameters: 1400 mV, 10 ms, and three pulses. Approximately 1 µg of PB transposase, *rtTA*, *Pax2*, *Eya1*, *Six1*, *Hoxa11*, *Hoxc11*, and *Hoxd11* PB vectors were transfected into ESCs. Transfected ESCs were cultured under standard mouse ESC conditions. One day after transfection, puromycin (1 µg/ml) was added to culture for 24 h to select for cells containing the transgenes. The cells that survived puromycin selection were allowed to grow and form ESC colonies.

Twenty-four clones were picked and expanded on gelatin- and MEF-coated plates and cultured under standard mouse ESC conditions. Genomic DNA was isolated from all 24 clones for genotyping purpose. Primers used to detect transgenes were as follows: *Pax2* (F: CCATACAGCCATCCCCAGTAC, R: TCACCACTTTGTACAAGAAAGCTG), *Eya1* (F: GGAGATGGTGTGGAAGAAGAGC, R: TCACCACTTTGTACAAGAAAGCTG), *Six1* (F: CAGAACTCGGTCCTTCTGCTC, R: TCACCACTTTGTACAAGAAAGCTG), *Hoxa11* (F: GCTGGAGCGAGAGTTCTTCTTC, R: TCACCACTTTGTACAAGAAAGCTG), *Hoxc11* (F: CGGATGCTGAACCTGACAGAC, R: TCACCACTTTGTACAAGAAAGCTG), and *Hoxd11* (F: GACTCCAACTCTCTCGGATGCT, R: TCACCACTTTGTACAAGAAAGCTG).

Ten clones containing all six transgenes (clones 6, 7, 9, 10, 11, 12, 19, 20, 23, and 24) were selected for quantitative PCR to evaluate transgene expression with and without DOX (1.5 µg/ml). RNA was isolated and quantitative PCR was performed. Primers used were as follows: *Pax2* (F: AAGCCCGGAGTGATTGGTG, R: CAGGCGAACATAGTCGGGTT), *Eya1* (F: CATAGCCGACTGAGTGGTAGT, R: GCTCTGTTTTAACTTCGGTGCC), *Six1* (F: ATGCTGCCGTCGTTTGGTT, R: CCTTGAGCACGCTCTCGTT), *Hoxa11* (F: TTTGATGAGCGTGGTCCCTG, R: AGGAGTAGGAGTATGTCATTGGG), *Hoxc11* (F: TCCAACCTCTATCTGCCCAGT, R: CAAGACGAGTAGCTGTTCCGA), *Hoxd11* (F: AAAAGACTCCAACTCTCTCGGA, R: AGACGGTCCCTGTTCAGTTTC), *β-actin* (F: GGCTGTATTCCCCTCCATCG, R: CCAGTTGGTAACAATGCCATGT), and *Gapdh* (F: AGGTCGGTGTGAACGGATTTG, R: TGTAGACCATGTAGTTGAGGTCA). DNA was amplified under the following conditions: 95 °C for 15 s, followed by 40 cycles of 95 °C for 5 s, and 60 °C for 30 s. The expression was normalized to *β-actin* and *Gapdh*. The three most DOX-responsive clones (clones 11, 19, and 23) were selected for differentiation (Supplementary Table II).

### Differentiating transgenic mouse ESC lines to renal progenitors

Transgenic mouse ESC lines (clones 11, 19, and 23) were differentiated to renal progenitors following our differentiation protocol. DOX (1.5 µg/ml) was added days 6–11, 11–16, or 16–21 of differentiation, which coincided with the progenitor induction stage of differentiation. Differentiation with no DOX was also performed as a control. RNA was collected at the end of differentiation, cDNA was synthesized, and progenitor marker expression was quantified using quantitative PCR.

### Organoid culture

At the end of the 21-day mouse kidney differentiation protocol, renal progenitors were dissociated into single cells and centrifuged at 200 × *g* at 10 °C for 5 min. The supernatant was discarded and the cell pellet was resuspended in 15% FBS Complete Media (DMEM supplemented with 15% FBS, 50 U/ml penicillin, 50 µg/ml streptomycin, 2 mM GlutaMAX, 0.1 mM NEAA, 1 mM sodium pyruvate, and 0.1 mM 2-mercaptoethanol) at a concentration of 35,000–50,000 cells/µl. A 10 µl droplet of cells was transferred onto a porous membrane filter (Whatman, #110407) to form a cell aggregate. Cell aggregates were cultured overnight in 15% FBS Complete Media containing with 10 µM Y27632. The next day, cell aggregates were then cultured for 24 h in 15% FBS Complete Media containing 3 µM CHIR99021 and 10 ng/ml Bmp7. Finally, cell aggregates were cultured in 15% FBS Complete Media alone for 6 days (8D organoids), 12 days (14D organoids), or 19 days (21D organoids). Unless stated otherwise, 8D organoids were used in experiments.

### Immunohistochemistry

Tissues were fixed in 10% NBF, dehydrated, and embedded in paraffin in the following manner: 80% ethanol for 30 min, 95% ethanol for 45 min, 100% for 2 h, toluene for 2 h, and paraffin for 2–3 h. Tissue blocks were sectioned, and tissue sections (5 µm thick) were dewaxed and immunostained. Briefly, a blocking buffer (PBS containing 0.1% Triton X-100 and 10% FBS) was applied onto dewaxed tissue sections for 1 h at RT. After blocking, primary antibodies (diluted 1:200) were added and incubated with tissue sections overnight at 4 °C. The next day, tissue sections were washed 4× for 10 min with PBS containing 0.1% Triton X-100 and incubated with secondary antibodies (diluted 1:500) for 1 h in the dark and at RT. Finally, tissues sections were washed 5× for 10 min with PBS containing 0.1% Triton X-100, stained with DAPI, and cover-slipped. The primary antibodies used were as follows: N-cadherin (Abcam, AB98952), cytokeratin 8 (Novus Biologicals, NB120-9287), E-cadherin (R&D Systems, AF748), pan-cytokeratin (Sigma, C2562), collagen IV (Abcam, AB6586), laminin (Abcam, AB11575), fibronectin (Abcam, AB23750), Pbx1 (Cell Signaling, 4342 S), aquaporin 2 (Alomone Labs, AQP002). The secondary antibodies used were as follows: anti-goat IgG 594, anti-mouse IgG 488, and anti-rabbit IgG Cy5.

### Whole-mount staining

Tissues were fixed in 10% NBF for 1–2 h at RT. Fixed tissues were washed three times with PBS containing 0.1% Triton X-100 and incubated in blocking buffer (PBS containing 0.1% Triton X-100 and 10% FBS) for 2–3 h at RT. After blocking, tissues were washed three times in PBS containing 0.1% Triton X-100 and incubated with primary antibodies (diluted 1:200) overnight at 4 °C. Tissues were then washed 4× for 30 min with PBS containing 0.1% Triton X-100. Secondary antibodies (diluted 1:500) were added for 2–4 h at RT. Finally, tissues were washed 4× for 30 min with PBS containing 0.1% Triton X-100, and stained with DAPI. The primary antibodies used were as follows: LTL (Vector Laboratories, B-1325), E-cadherin (Abcam, AB11512), and Wt1 (Abcam, AB89901). The secondary antibodies used were as follows: strepavidin-Cy5, anti-rat IgG 594, and anti-rabbit IgG 488.

## Supplementary information

supplementary-materials

reporting-summary

## Data Availability

Gene expression (microarray) datasets generated in this study are available in the Gene Expression Omnibus (GEO) repository (GEO accession number: GSE140012).
