# Supplementary material for: Recapitulating kidney development in vitro by priming and differentiating mouse embryonic stem cells in monolayers
Source: NPJ Regen Med. 2020 Apr 20;5:7. doi: 10.1038/s41536-020-0092-5 (PMC7171095; doi:10.1038/s41536-020-0092-5)
Supplement: Supplementary file 1 — supplementary-materials [file 41536_2020_92_MOESM1_ESM.pdf]

Supplementary Figures and Tables

Supplementary Figure 1: Six2, Pax2 and Wt1 expression in embryonic kidney cells and undifferentiated embryonic stem cells (ESC).

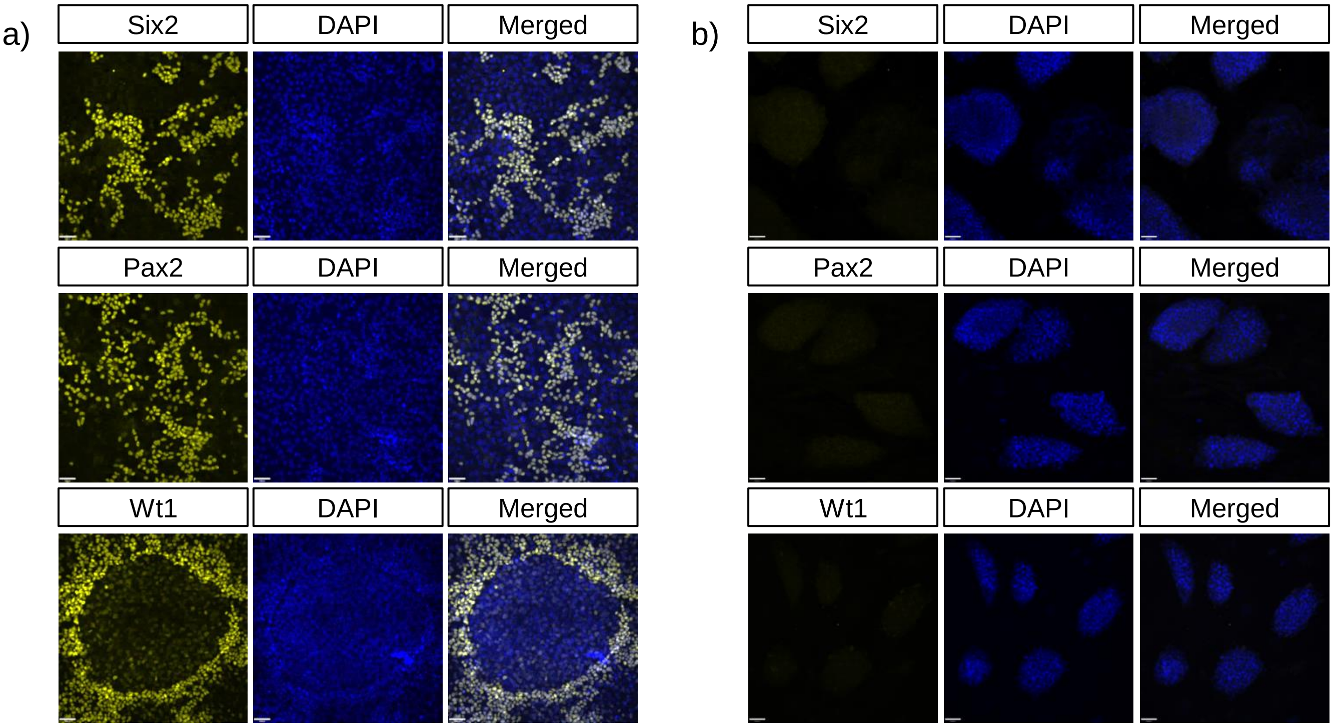

a) Immunocytochemistry shows that E13.5 embryonic kidney cells express Six2, Pax2 and Wt1. b) Immunocytochemistry shows that undifferentiated 1B10 embryonic stem cells do not express Six2, Pax2 and Wt1. Scale bar = 40 μm.

Supplementary Figure 2: Secondary antibody controls for differentiated renal progenitors, embryonic kidney cells and undifferentiated mouse embryonic stem cells (ESC).

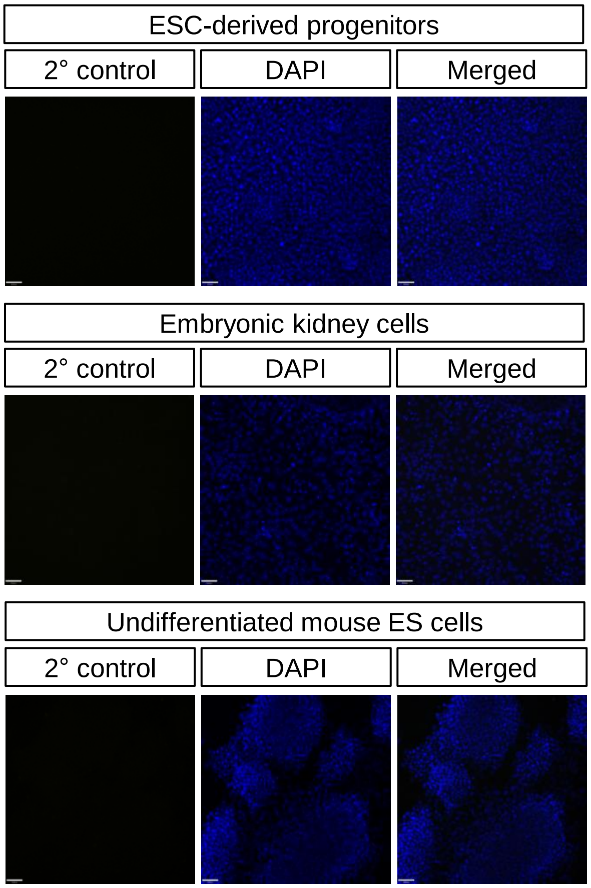

Control experiments for immunocytochemistry show that secondary antibodies do not bind non-specifically to cells. Scale bar = 40 μm.

Supplementary Figure 3: Expression of pluripotency-associated markers in renal progenitors.

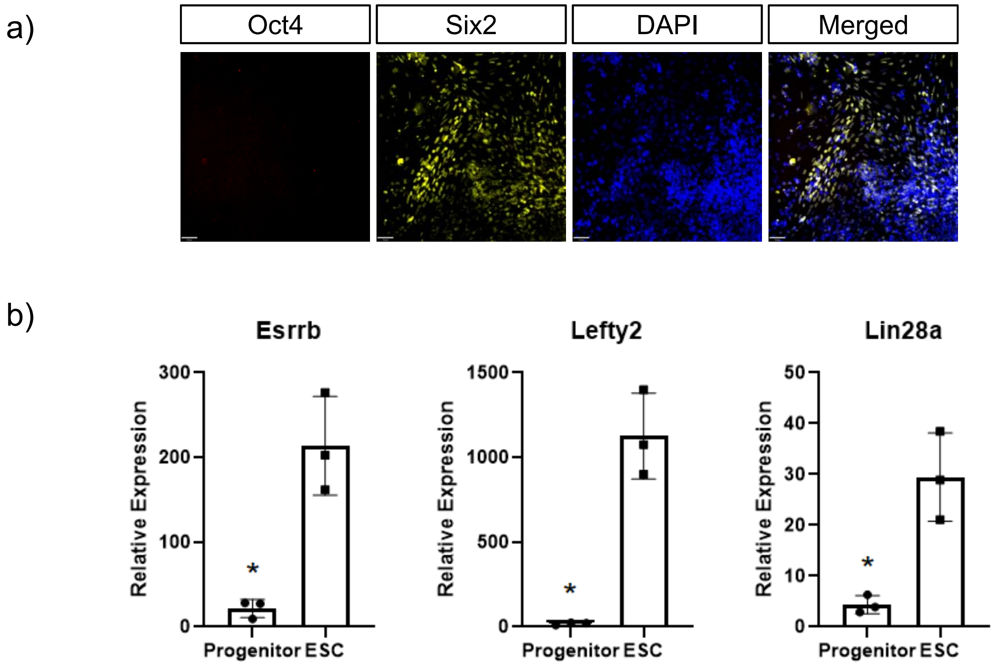

a) Immunocytochemistry shows the downregulation of Oct4 in renal progenitors. Scale bar = 40 µm. b) Quantitative PCR demonstrates the downregulation of Esrrb, Lefty2 and Lin28a. Gene expression is relative to E13.5 embryonic kidney. Asterisk (*) indicates that expression is significantly different ($p \leq 0.05$) from embryonic stem cells (ESC), as determined by t-test with Welch’s correction. n = 3. Error bars represent standard deviation.

Supplementary Figure 4: Principle component analysis (PCA) plots.

a)

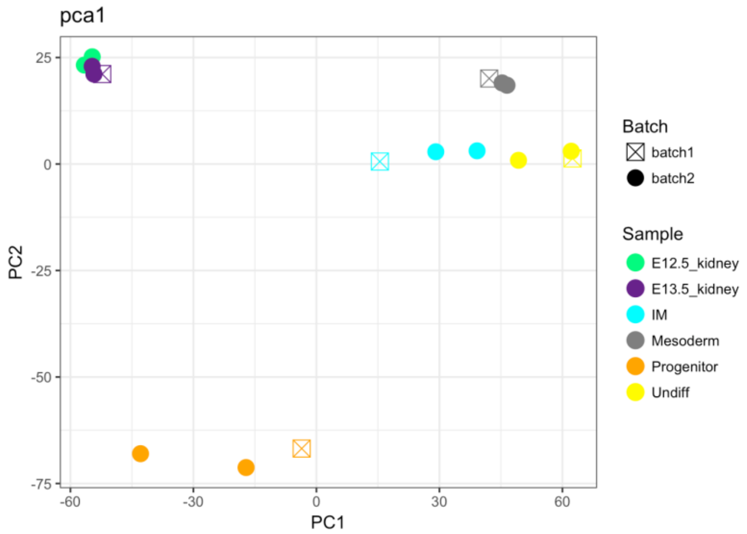

b)

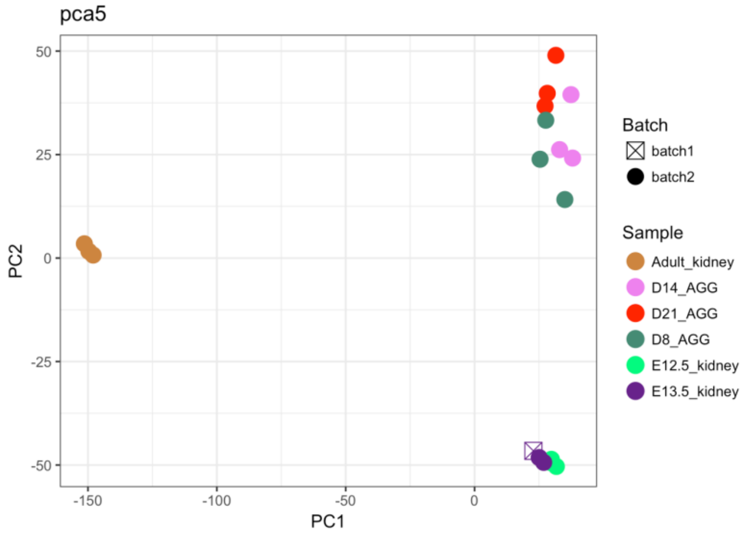

a) PCA plot of undifferentiated mouse embryonic stem cells (ESC), mesoderm cells, intermediate mesoderm cells, progenitor cells, and mouse embryonic kidney. Principle component one (PC1; 43.2% variance) separates embryonic kidneys and renal progenitors from embryonic stem cells, mesoderm cells and intermediate mesoderm cells. PC2 (20.5% variance) separates renal progenitors from embryonic kidney, embryonic stem cell, mesoderm and intermediate mesoderm. b) PCA plot of kidney organoids cultured in vitro for 8, 14 and 21 days. Principle component one (PC1; 59.1% variance) separates 8D organoid, 14D organoid, 21D organoid and embryonic kidney from adult kidney, while PC2 (17.7% variance) separates 8D organoid, 14D organoid, 21D organoid and adult kidney from embryonic kidney. The colours represent different samples (n = 3) and the shapes represent different microarray batches.

Supplementary Figure 5: Kidney organoids formed from embryonic kidney cells and embryonic stem cell (ESC)-derived renal progenitors.

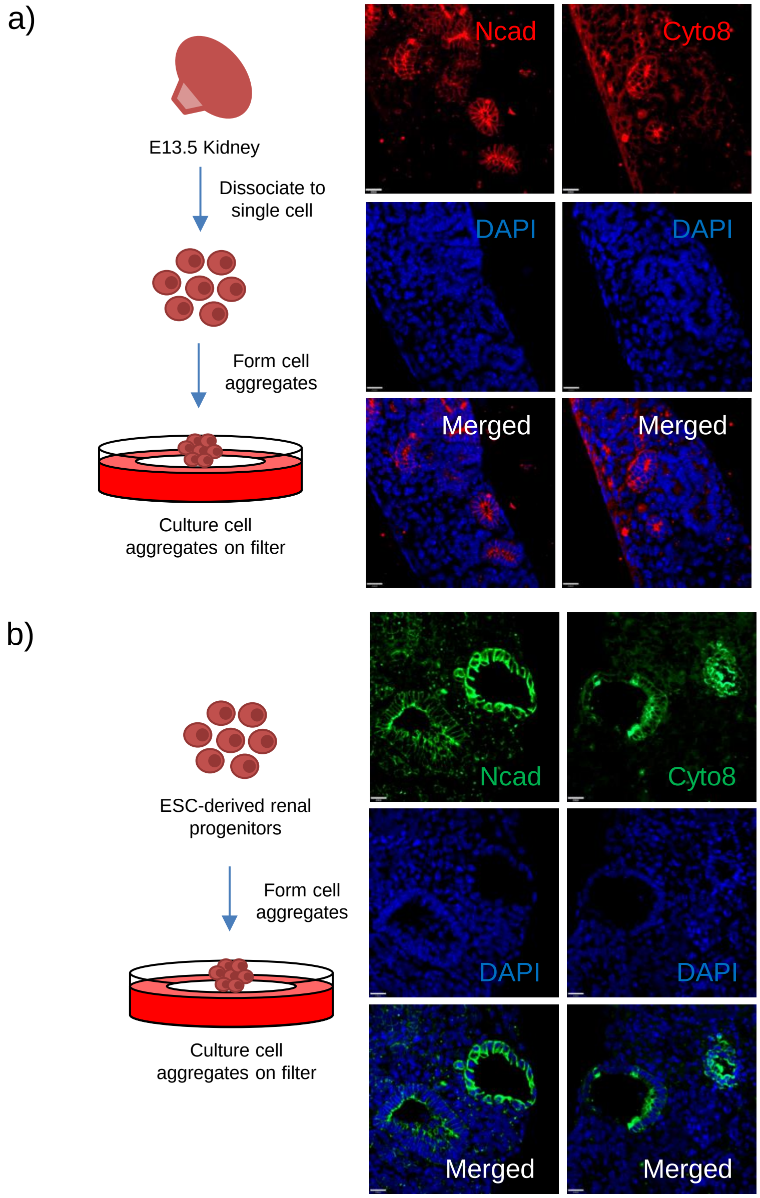

a) The dissociation and re-aggregation of E13.5 embryonic kidney cells gives rise to kidney organoids that contain N cadherin (Ncad)- and Cytokeratin 8 (Cyto8)-positive tubules. b) Aggregating and culturing ESC-derived renal progenitors also gives rise to kidney organoids containing N cadherin (Ncad)- and Cytokeratin 8 (Cyto8)-positive tubules. Scale bar = 20 μm.

Supplementary Figure 6: Pbx1+ stromal cells and Aquaporin 2+ collecting duct cells in kidney organoids formed from embryonic stem cell (ESC)-derived renal progenitors.

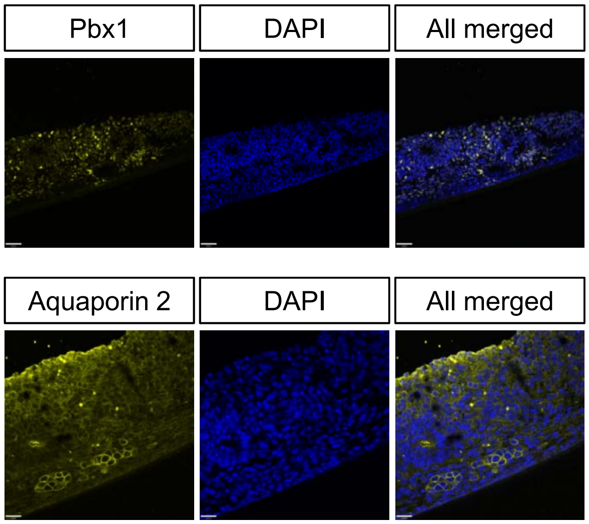

Top panel: Pbx1+ stromal cells intercalated in the space between tubules. Bottom panel: Presence of Aquaporin 2+ collecting duct cells in kidney organoids. Scale bar = 20 µm.

Supplementary Figure 7: Expression of extracellular matrix proteins, E cadherin and Cytokeratin in cell aggregates made from embryonic stem cells (ESC).

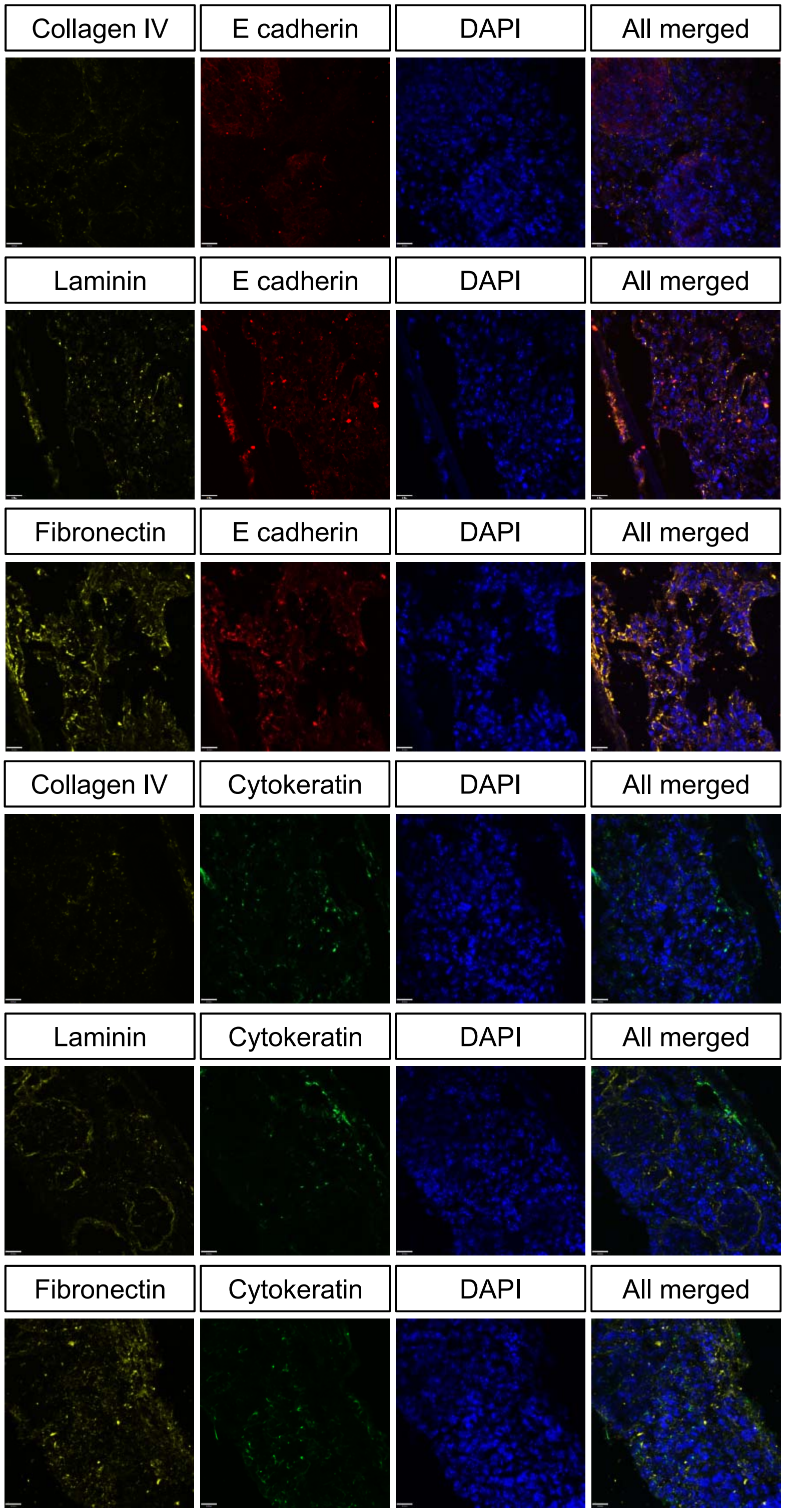

Immunohistochemistry of cross sections of cell aggregates made from undifferentiated ESC shows the absence of E cadherin, Cytokeratin and extracellular matrix protein expression. Scale bar = 20 μm.

Supplementary Figure 8: Secondary antibody controls for kidney organoids and cell aggregates made from embryonic stem cells (ESC).

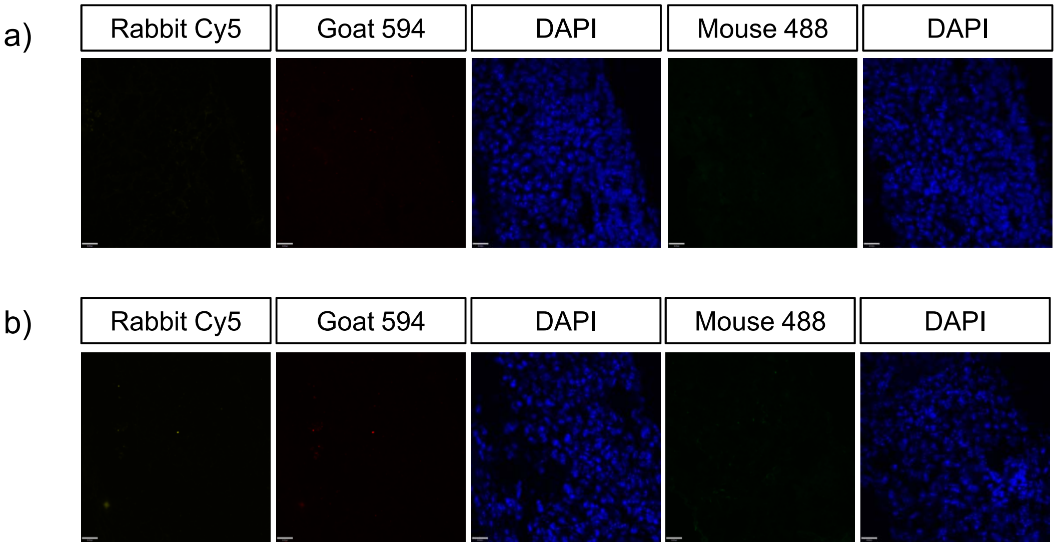

a) Secondary antibodies do not bind non-specifically to kidney organoids. b) Secondary antibodies do not bind non-specifically to cell aggregates made from ESC. Scale bar = 20 μm.

Supplementary Table I: The presence or absence of transgenes in 24 embryonic stem cell (ESC) clones.

	Pax2	Eya1	Six1	Hoxa11	Hoxc11	Hoxd11
1	Weak	Weak	Weak	Weak	Weak	Weak
2	Weak	Weak	+	+	+	+
3	Weak	Weak	+	+	+	+
4	Weak	+	+	+	+	+
5	+	Weak	+	+	+	+
6	+	+	+	+	+	+
7	+	+	+	+	+	+
8	Weak	Weak	Weak	+	+	+
9	+	+	+	+	+	+
10	+	+	+	+	+	+
11	+	+	+	+	+	+
12	+	+	+	+	+	+
13	+	+	+	+	+	+
14	Weak	+	+	+	+	+
15	Weak	Weak	+	+	+	+
16	Weak	+	+	+	+	+
17	-	-	+	+	+	+
18	Weak	-	+	+	+	+
19	+	+	+	+	+	+
20	+	+	+	+	+	+
21	-	-	-	-	-	Weak
22	Weak	Weak	+	+	+	+
23	+	+	+	+	+	+
24	+	+	+	+	+	+

Supplementary Table II: Transgene expression levels in the selected ten transgenic mouse embryonic stem cell (ESC) clones.

		Pax2	Eya1	Six1	Hoxa11	Hoxc11	Hoxd11
Clone 6	No DOX	0.01384	0.00362	7.50045	0.01191	0.1618	0.00414
	DOX	0.14819	0.00914	97.9678	0.21807	17.9981	0.17836
Clone 7	No DOX	0.01308	0.05464	36.0641	0.01182	0.73242	0.00612
	DOX	0.08638	0.03968	602.444	0.39896	32.433	0.61297
Clone 9	No DOX	0.01718	0.00829	4.15085	0.01452	0.3946	0.04224
	DOX	0.00926	0.01173	573.363	0.33375	56.377	0.92331
Clone 10	No DOX	0.00644	0.01502	4.1692	0.00999	0.74904	0.01149
	DOX	0.30604	0.04396	580.779	0.5515	31.1384	0.83778
Clone 11	No DOX	0.0585	0.02942	35.1211	0.03499	0.83594	0.02527
	DOX	2.31736	0.24091	4540.02	1.44849	521.091	9.81717
Clone 12	No DOX	0.0033	0.00999	0.2619	0.00585	0.04661	0.00411
	DOX	0.01131	0.01312	5.16268	0.01284	0.19011	0.04876
Clone 19	No DOX	0.02415	0.00323	4.40303	0.01448	0.37911	0.00829
	DOX	4.07795	0.04866	1973.66	3.87032	319.366	2.01409
Clone 20	No DOX	0.06873	0.01978	24.3738	0.02419	0.54976	0.02307
	DOX	0.71499	0.13292	1713.79	0.88279	317.675	7.85788
Clone 23	No DOX	0.02963	0.01848	16.9319	0.04009	3.71823	0.02819
	DOX	3.8507	0.24827	491.38	7.75655	643.81	8.88231
Clone 24	No DOX	0.0207	0.00768	15.6026	0.05762	0.75423	0.00586
	DOX	0.06029	0.01294	18.5375	0.11245	2.01272	0.02382
Wildtype	No DOX	0.01775	0.00254	2.399	0.00767	0.08696	0.00341
	DOX	0.00819	0.00768	0.81095	0.00594	0.05676	0.00624

Supplementary Figure 9: Six2 expression in expanded embryonic stem cell (ESC)-derived renal progenitors.

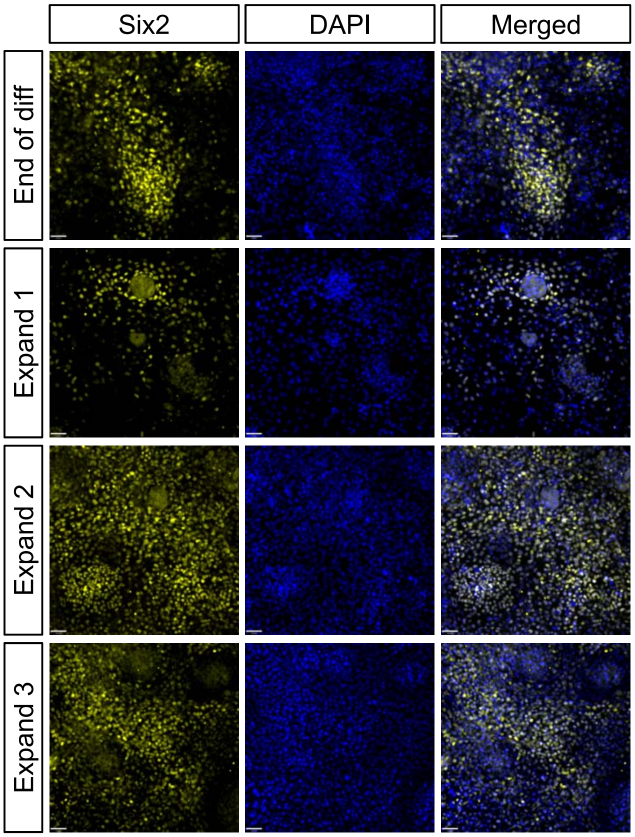

Immunocytochemistry shows that ESC-derived renal progenitors can be expanded in culture and maintain Six2 expression. Scale bar = 40 μm.

Supplementary Figure 10: Pax2 expression in expanded embryonic stem cell (ESC)-derived renal progenitors.

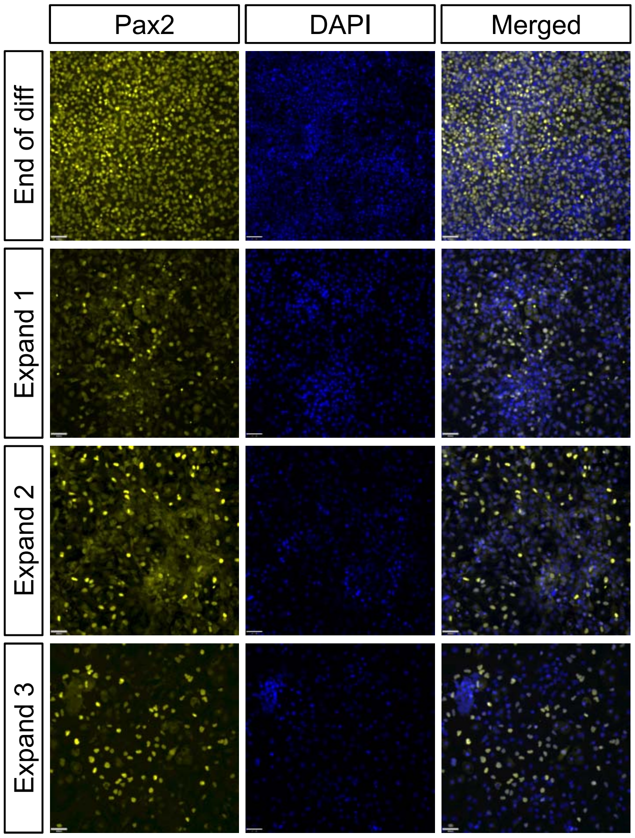

Immunocytochemistry shows that ESC-derived renal progenitors can be expanded in culture and maintain Pax2 expression. Scale bar = 40 μm.

Supplementary Figure 11: Wt1 expression in expanded embryonic stem cell (ESC)-derived renal progenitors.

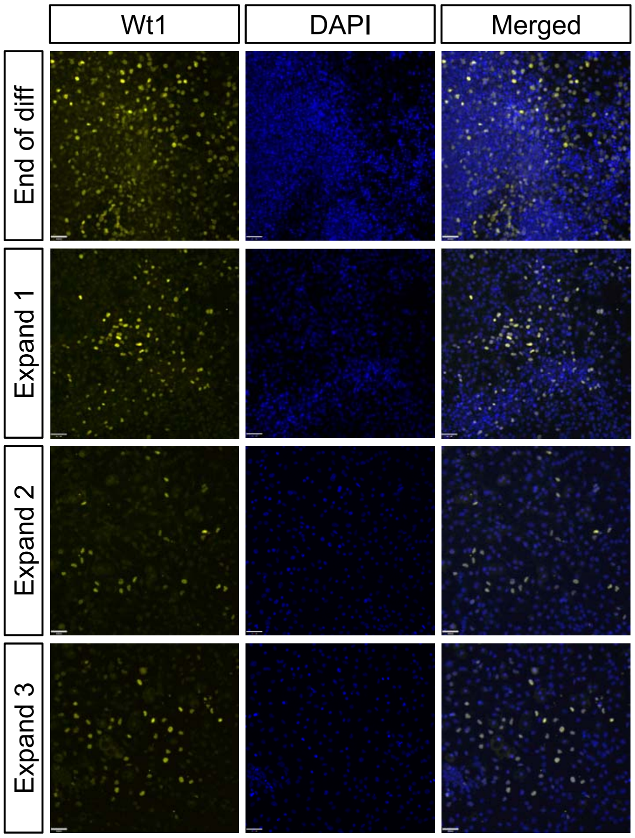

Immunocytochemistry shows that ESC-derived renal progenitors can be expanded in culture and maintain Wt1 expression. Scale bar = 40 μm.
